# Signatures of Co-evolution and Co-regulation in the CYP3A and CYP4F Genes in Humans

**DOI:** 10.1093/gbe/evad236

**Published:** 2024-01-11

**Authors:** Alex Richard-St-Hilaire, Isabel Gamache, Justin Pelletier, Jean-Christophe Grenier, Raphaël Poujol, Julie G Hussin

**Affiliations:** Département de biochimie et médecine moléculaire, Université de Montréal, Montreal, QC, Canada; Sainte-Justine Hospital, Research Center, Montreal, QC, Canada; Département de biochimie et médecine moléculaire, Université de Montréal, Montreal, QC, Canada; Montreal Heart Institute, Research Center, Montreal, QC, Canada; Département de biochimie et médecine moléculaire, Université de Montréal, Montreal, QC, Canada; McGill CERC in Genomic Medicine, McGill University, Montreal, Canada; Montreal Heart Institute, Research Center, Montreal, QC, Canada; Montreal Heart Institute, Research Center, Montreal, QC, Canada; Montreal Heart Institute, Research Center, Montreal, QC, Canada; Département de médecine, Université de Montréal, Montreal, QC, Canada; Mila-Quebec AI institute, Montreal, QC, Canada

**Keywords:** Cytochromes P450, population genetics, linkage disequilibrium, co-evolution, gene expression

## Abstract

Cytochromes P450 (CYP450) are hemoproteins generally involved in the detoxification of the body of xenobiotic molecules. They participate in the metabolism of many drugs and genetic polymorphisms in humans have been found to impact drug responses and metabolic functions. In this study, we investigate the genetic diversity of *CYP450* genes. We found that two clusters, *CYP3A* and *CYP4F*, are notably differentiated across human populations with evidence for selective pressures acting on both clusters: we found signals of recent positive selection in *CYP3A* and *CYP4F* genes and signals of balancing selection in *CYP4F* genes. Furthermore, an extensive amount of unusual linkage disequilibrium is detected in this latter cluster, indicating co-evolution signatures among *CYP4F* genes. Several of the selective signals uncovered co-localize with expression quantitative trait loci (eQTL), which could suggest epistasis acting on co-regulation in these gene families. In particular, we detected a potential co-regulation event between *CYP3A5* and *CYP3A43*, a gene whose function remains poorly characterized. We further identified a causal relationship between *CYP3A5* expression and reticulocyte count through Mendelian randomization analyses, potentially involving a regulatory region displaying a selective signal specific to African populations. Our findings linking natural selection and gene expression in *CYP3A* and *CYP4F* subfamilies are of importance in understanding population differences in metabolism of nutrients and drugs.

**Significance**

Genetic diversity in Cytochromes P450 enzymes has been hypothesized to evolve subjected gene–gene interactions, potentially explaining their tendency of living in clusters within genomes. Here, we confirmed outstanding selective signatures in the *CYP3A* and *CYP4F* gene clusters, and identified a high level of unusual correlation between genetic markers far apart from each other, suggesting selection on combinations of genotypes in distinct regions, a signature of co-evolution due to epistasis.

## Introduction

In the last decades, it has become clear that every individual has their own “fingerprint” of alleles encoding drug-metabolizing enzymes, playing central roles in the metabolism of endogenous and exogenous compounds. It was established that hydrophobic molecules are first modified by oxidation and subsequently excreted as water-soluble forms, two distinct steps now described as phases I and II. Phase I is performed mainly by Cytochromes P450 (CYP450) enzymes, able to catalyze a considerable variety of oxidation reactions for many structural classes of chemicals (including the majority of drugs) (Danielson 2002; Nebert and Dalton 2006). They metabolically activate parent compounds to electrophilic intermediates, while phase II enzymes conjugate these intermediates towards more easily excretable derivatives.

*CYP450* genes are a super-family of genes which appeared more than 3.5 billion years ago (Wright et al. 2019), being present in fungi, plants, bacteria, and animals. Genes are grouped into families and subfamilies based on sequence similarity: genes from the same family have sequence similarity greater than 40% and, to be grouped into a subfamily, their sequence similarity must be greater than 55% (Nelson et al. 1996).

In humans, the *CYP450* family includes 57 genes and 58 pseudogenes (Nelson et al. 2004) grouped in 18 families (Nebert et al. 2013). Several *CYP450* genes are found in clusters in the human genome but some members of the subfamilies can be spread out across the genome. For example, the CYP4F subfamily has genes on chromosome 19 and pseudogenes on multiple chromosomes. The *CYP2D6* gene is the most widely studied *CYP450* gene in humans, due to its role in the metabolism of many drugs (Gaedigk 2013; Gaedigk et al. 2017) along with *CYP3A4* and *CYP3A5*, members of the *CYP3A* subfamily (Wang et al. 2011; Elens et al. 2012; Lamba et al. 2012; Tavira et al. 2013; Rojas et al. 2015). However, not all *CYP450* genes or families have been studied thoroughly, and details on the evolution and clinical significance are lacking for several families, such as the *CYP4F* subfamily.

Several *CYP450* genes have been suggested to have undergone natural selection in humans (Carlson et al. 2005; Voight et al. 2006). Other studies of the genetic diversity for specific *CYP450* subfamilies in human populations confirmed the presence of positive (Qiu et al. 2008; Bains et al. 2013), balancing (Janha et al. 2014), or purifying selection signatures (Yasukochi and Satta 2015). One example is *CYP2C19*, involved in the metabolism of clopidogrel (Scott et al. 2013; Brown and Pereira 2018), where signals of positive selection on its alleles conferring slow metabolism (CYP2C19*2 and CYP2C19*3) were detected using relative extended haplotype homozygosity (REHH) (Janha et al. 2014). CYP2C19*2 is detected worldwide, but CYP2C19*3 is only present in people of Asian descent. The selective advantages may have been caused by diet and environmental pollutants impacting humans over thousands of years and could differ between ethnic groups. Additionally, low $F_{ST}$ values across *CYP2C19* SNPs suggest balancing selection in *CYP2C19* (Janha et al. 2014). The excess of alleles at intermediate frequencies could reflect the evolution of balanced polymorphisms, which is to be expected in evolutionarily old enzymes responsible for numerous critical life functions.

Moreover, the detection of natural selection signals in the *CYP450* genes raises the possibility that the selective advantage acts on polymorphisms that modulate gene expression, widely known as expression quantitative trait loci (eQTL) (Kudaravalli et al. 2009). Detecting eQTLs linked to selection signals helps clarifying how gene expression is regulated and can lead to a better understanding of variants’ biological effects (Nica and Dermitzakis 2013). Furthermore, analyzing eQTLs helps in the detection of gene–gene interaction (Huang et al. 2013) and co-regulation between genes (Lehner 2011). Such gene–gene interactions can also be detected by looking at patterns of linkage disequilibrium (LD), as evolution will maintain co-evolving polymorphisms on the same haplotypes (Rohlfs et al. 2010), which can also be detected as balancing selection signatures.

Here, we investigated genetic diversity and selective pressures across human populations in *CYP450* genes. Two subfamilies emerged from our analyses and were investigated in greater depth: the *CYP3A* and *CYP4F* families. Both subfamilies were generated by duplication events resulting in consecutive genes in the same genomic region, or gene cluster (supplementary fig. S1, Supplementary Material online). The CYP3A subfamily contains four genes and four pseudogenes located in a genomic region of about 220 KB on chromosome 7. They metabolize around 50% of common drugs. The CYP4F subfamily has six genes located in a genomic region of about 430 KB on chromosome 19 and have mostly been associated with metabolism of lipids. We found that both families exhibit selective pressures in human populations and that the SNPs under selection could impact gene expression levels in several tissues. Furthermore, our results suggest interactions between the genes in both *CYP450* subfamilies, providing evidence of co-evolution and co-regulation within these gene clusters, that may vary between populations.

## Results

We obtained genotypic data from the 1000 Genomes project phase 3 release (1000G) (Genomes Project Consortium et al. 2015). A total of 2,157 individuals were analyzed from 22 populations, which belongs to four of the five super-populations included in the project (i.e. Africa, Europe, East Asia, and South Asia).

### Global Genetic Diversity Across Populations in CYP450 Genes

First, we aimed to identify global genetic patterns by calculating Tajima’s D values for each *CYP450* genes in each population of the 1000G dataset to provide insights into the non-neutral forces that act on these genes. A total of 61,739 biallelic SNPs were analyzed in all of the 57 *CYP450* genes, and for each gene, we computed the mean Tajima’s D per gene and also in 1 Kb windows. Significantly low Tajima’s D values indicate an excess of rare alleles, whereas significantly high values of Tajima’s D suggest an excess of intermediate frequency alleles, which can reflect the occurrence of balancing selection.

**Fig. 1.**
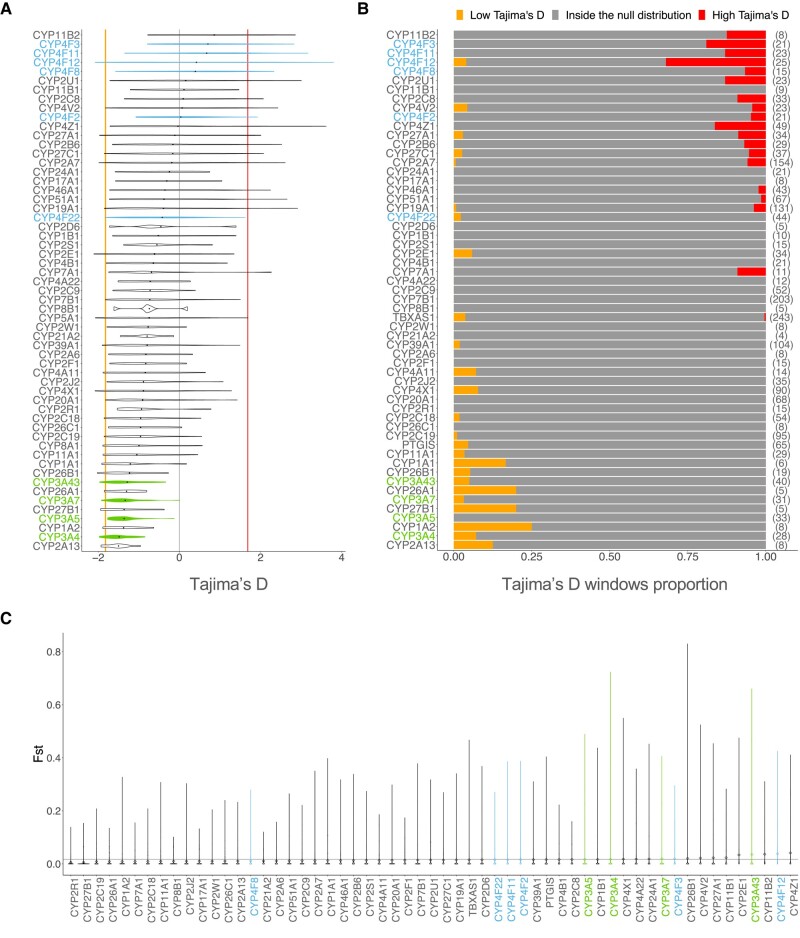
Metrics of diversity and differentiation among *CYP* genes. a) Distribution of Tajima’s D values computed on windows of 1 Kb for each CYP450 genes in the European populations. The 2.5th percentile is marked by the orange vertical line on the left, and the 97.5th percentile is marked by the red vertical line on the right, representing the significance threshold. b) Proportion of Tajima’s D windows lying outside the null distribution for each CYP450 gene. For each gene, the total number of windows of Tajima’s D is shown beside the proportions, between brackets. The windows with Tajima’s D values below the 2.5th percentile is displayed in orange on the left side of the plot and over the 97.5th percentile is displayed in red on the right side of the plot. c) Distribution of $F_{ST}$ values for each CYP450 gene calculated on 4 super-populations (AFR, EUR, EAS, and SAS). The mean $F_{ST}$ of chromosome 22, the null distribution, is displayed with the gray horizontal line.

In European populations, nine genes had Tajima’s D values consistently below 0 (Fig. 1a). We assessed significance based on the empirical (null) distribution, which allows to determine whether any genes have values that are higher or lower than expected while taking population-specific demographic factors into account (see Materials and Methods). The proportion of 1 Kb-windows of each gene lying outside the null distribution is shown in Fig. 1b. *CYP26A1*, *CYP27B1*, and *CYP1A2* had the largest proportion of windows with significantly low D values; however, these genes are quite small (4.4, 4.9, and 7.8 Kb, respectively), meaning that the signal is driven by one or two windows only. Interestingly, the four *CYP3A* genes in our dataset were all included in this group of nine genes, suggesting that strong purifying selection pressures may be acting, however complete selective sweeps driven by positive selection can also create this lack of diversity (Kim and Stephan 2002). Notably, *CYP3A5* has a low Tajima’s D average but no 1 Kb-window is significantly lower than expected, whereas other *CYP3A* genes have several windows showing significantly low Tajima’s D values. All *CYP450* genes show negative Tajima’s D values, as expected in coding regions, but ten genes have a mean above 0, which suggests relaxation of purifying selection pressure. The presence of several 1 Kb-windows significantly enriched for high D values can also reflect the presence of localized balancing selection signatures within these genes. Of these ten genes, five are in the CYP4F subfamily: *CYP4F3*, *CYP4F11*, *CYP4F12*, *CYP4F8*, and *CYP4F2*. The strongest of these signals is seen on *CYP4F12* (Fig. 1b). Interestingly, the only CYP4F gene that does not show this specific signature is *CYP4F22*, which is the ancestral gene of the *CYP4F* cluster (Kirischian and Wilson 2012). Notably, these analyses were also performed for each of the subpopulations, yielding similar results (supplementary fig. S2, Supplementary Material online).

Because population differentiation can also help identifying natural selection signatures within genes, we calculated the mean fixation index ($F_{ST}$) across *CYP450* genes (Materials and Methods). $F_{ST}$ measures the differentiation between populations using genotype frequencies, with high $F_{ST}$ values indicating that the average pairwise heterozygosity is higher between than within populations. Figure 1c shows the distribution of $F_{ST}$ values for each *CYP450* gene calculated on 4 super-populations (AFR, EUR, EAS, and SAS). CYP4F genes are scattered across the *CYP450* spectrum, with *CYP4F12* having the second highest mean $F_{ST}$ while *CYP4F8* is in the bottom half of the distribution. Mean $F_{ST}$ of genes of the *CYP3A* subfamily are in the highest values, meaning that these genes have a high divergence between population’s genotype frequencies. This could indicate that the low Tajima’s D in *CYP3A* reflects positive rather than extreme purifying selection.

### Positive Selection in CYP3A and CYP4F Subfamilies

The global neutrality and differentiation analyses of *CYP450* genes suggest that positive selection, either directional (*CYP3A*) or balancing (*CYP4F*), may be acting on subfamilies of *CYP450* genes, possibly in a concerted fashion. To further validate positive selection signatures and identify specific putative sites, we used the integrated haplotype score (iHS), which leverages linkage disequilibrium (LD) patterns in a specific population (Voight et al. 2006). Typically, an absolute value of iHS greater than 2 at a SNP suggests that the region around the SNP is under selection (Voight et al. 2006).

**Fig. 2.**
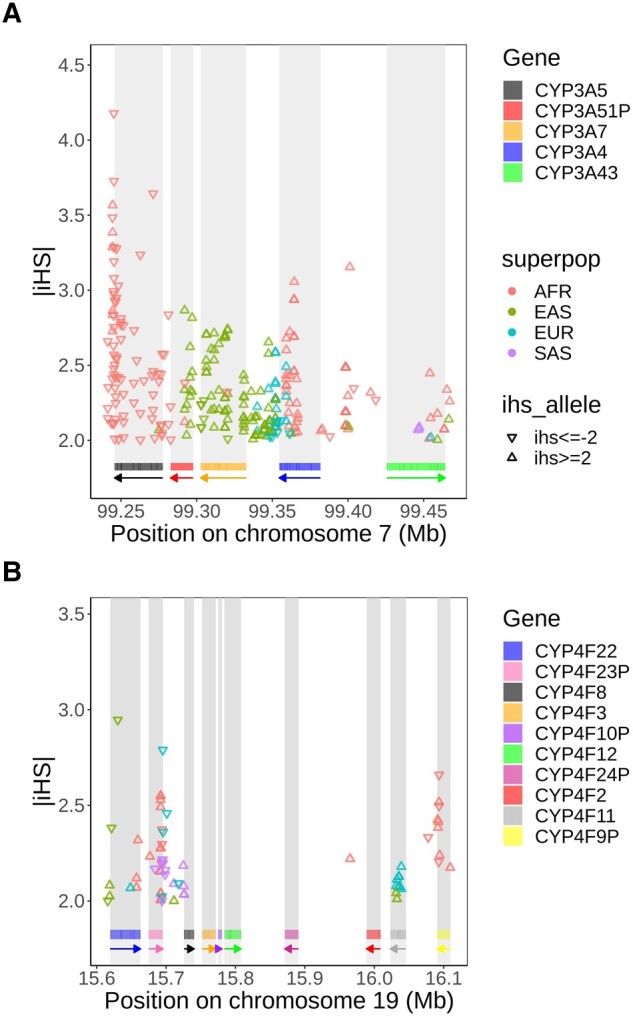
Distribution of SNPs with high |iHS| values (|iHS|≥2) in the a) CYP3A and b) CYP4F cluster. A triangle standing on its base means an iHS value ≥2, indicating that the ancestral allele has increased in frequency, and a triangle standing on its point means an iHS value ≤−2, indicating that the positive selection is acting on the derived allele. SNPs located in repetitive elements and sequences are masked. Rectangles below the plot show the position of each gene, and arrows indicate on which strand the gene is located.

In the *CYP3A* cluster, significant iHS values are detected (Fig. 2a), but signals of positive selection differ between populations. Many signals are detectable in Africans, in East Asians and in Europeans, while fewer signals are detectable in South Asians. Signals of positive selection are noticeable in *CYP3A5*, *CYP3A51P*, *CYP3A4* and *CYP3A43* among Africans. In particular, iHS values in *CYP3A5* are consistently below −2, indicating that the derived alleles have quickly increased in frequency, a signature of positive selection. Interestingly, unlike populations of European descent where the *CYP3A4* gene is typically the most expressed, the *CYP3A5* gene is the most expressed in the African individuals (Kuehl et al. 2001; Burk and Wojnowski 2004). Among East Asians, the selective sweep is located from *CYP3A51P* to *CYP3A4*, and among South Asians, in *CYP3A43*. Lastly, for Europeans, signals of positive selection are detectable in the region between *CYP3A7* and *CYP3A4*, a signal also present in the East Asian population. *CYP3A43* is the only gene with signals in all super-populations. With these results, we establish robust evidence for positive selection acting on *CYP3A* genes, corroborating and extending observations from previous studies (Thompson et al. 2004; Voight et al. 2006; Qiu et al. 2008; Li et al. 2011).

Positive selective pressure is also detected in the *CYP4F* cluster, but on a smaller scale. For the *CYP4F* cluster, signals of positive selection are visible in *CYP4F22*, *CYP4F23P*, *CYP4F11*, and *CYP4F9P* (Fig. 2b). The region between the pseudogene *CYP4F23P* and the gene *CYP4F8* also shows high iHS values, indicating positive selection in every super-population. iHS values greater than 2 are present in *CYP4F11* in Europeans and East Asians, indicating positive selection acting on ancestral alleles. *CYP4F9P* has significant iHS values in Africans. Again, most iHS values are greater than 2, indicating selective pressures on ancestral alleles, but the three strongest signals are seen for derived alleles (iHS below −2), suggesting these SNPs may be driving the signal.

### Balancing Selection in CYP3A and CYP4F Subfamilies

The Tajima’s D analyses (Fig. 1) suggested balancing selection in the *CYP4F* cluster. To confirm this finding, we used the Beta score (Siewert and Voight 2017), a statistic which detects clusters of alleles with similar allele frequencies, developed to specifically test whether balancing selection is present at specific loci.

**Fig. 3.**
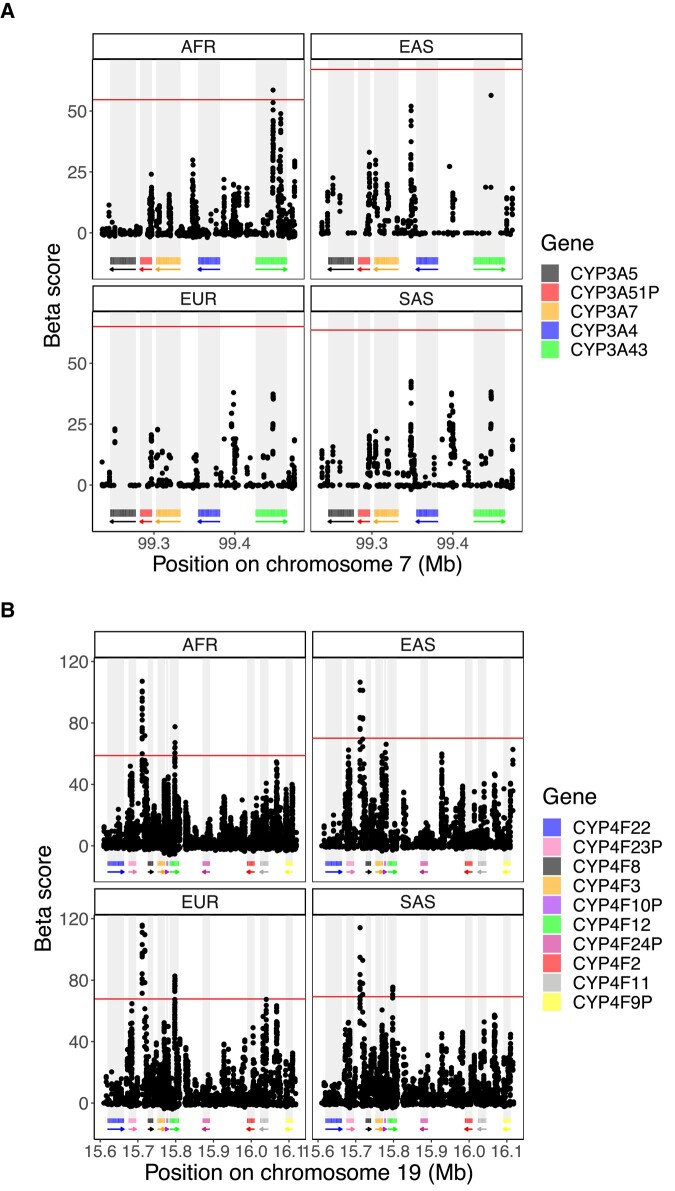
*β* score in the chromosomal region of the a) CYP3A and b) CYP4F cluster for the four super-populations analyzed. The *β* score was calculated on the 1000G dataset and the 99th percentile indicating the top 1% *β* score is displayed by the horizontal line in red. Rectangles below the plot show the position of each gene, and arrows indicate on which strand the gene is located.

We considered *β* score in the top 1% of the whole chromosome as significant *β* scores (empirical P−value<0.01), which can vary between populations. In contrast to iHS, very few significant *β* score values are seen in the *CYP3A* cluster. Only one SNP in *CYP3A43* meets this criteria in Africans, in the same region where balancing selection was also identified in a previous study (Aqil et al. 2023). The same signal can been seen in the other populations, but it is weaker and do not pass our 1% threshold (Fig. 3a). Overall, these results show no clear evidence of balancing selection acting on the *CYP3A* cluster. In line with Tajima’s D results, clearer signals are seen in the *CYP4F* cluster, which show larger *β* scores compared to the *CYP3A* cluster: the highest *β* score in the *CYP4F* cluster is almost twice as high as the highest *CYP3A*’s *β* score. SNPs in *CYP4F12* show highly significant *β* scores, replicated among Africans, Europeans and South Asians, but not in the East Asians. Also, the region between *CYP4F23P* and *CYP4F8* has the most extreme *β* score in the region, and the signal is visible in all super-populations (Fig. 3b). These consistent signals across populations provide convincing evidence of balancing selection acting around *CYP4F8* and *CYP4F12*. Weaker signals, which do not pass our significance threshold but are seen consistently between populations, are seen in *CYP4F23P* and *CYP4F11*. Taken together, these results demonstrate evidence supporting the presence of balancing selective pressures in the *CYP4F* cluster but show a lack of evidence for balancing selection across the *CYP3A* cluster.

### Detection of Unusual Linkage Disequilibrium

Since *CYP3A* and *CYP4F* genes are in a gene cluster and selective pressures are acting on these genes, co-evolution could be occurring. Indeed, the different combinations of alleles which co-occurred during evolution can lead to concerted selective pressure, or co-evolution, depending on the resulting fitness of the individuals (Rohlfs et al. 2010). Such co-evolution signals can be revealed by analyzing patterns of linkage disequilibrium (LD) beyond local associations due to allelic proximity, in order to detect whether specific combinations of alleles (or genotypes) at two distinct loci are particularly overrepresented. To do so, we calculated the genotyped-based LD ($r^{2}$) between each pair of SNPs with minor allele frequency (MAF) above 0.05 in the two CYP450 clusters, across each 1000G subpopulation (Materials and Methods). Under neutrality, the LD association between SNPs is expected to decrease as genetic distance between the SNPs increases, allowing us to build an empirical distribution by considering clusters of genes of similar size genome-wide (Materials and Methods) to the clusters under investigation. Pairs of SNPs showing unusual LD (uLD) values, lying outside of this null distribution, are therefore likely transmitted together more often than expected, making it possible to identify candidate sites that are co-evolving.

**Fig. 4.**
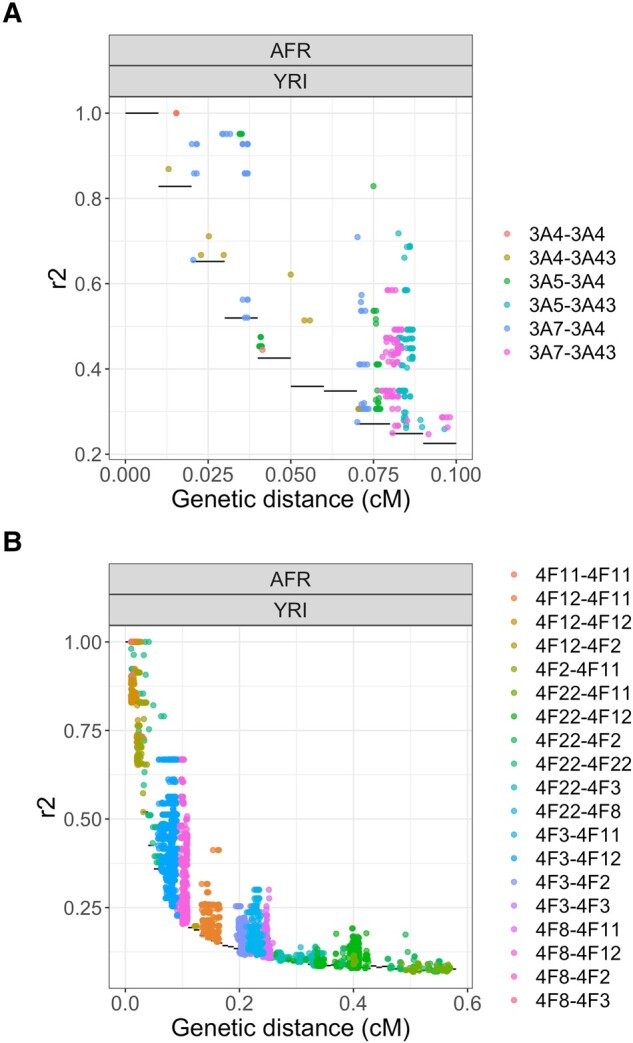
$r^{2}$ values between each pairs of SNPs in the a) CYP3A and b) CYP4F cluster in the Yoruba (YRI, AFR) population. The distance between the SNPs is in centimorgan (cM). Only $r^{2}$ values over the null distribution are shown. The null distribution is shown with black horizontal lines. Dots are colored according to which genes are involved in the pair.

In both clusters, strong signals of uLD are present (Fig. 4, supplementary fig. S3, Supplementary Material online) compared to matched gene clusters (Materials and Methods), with *CYP4F* showing much more extreme signals than *CYP3A* (8.1% vs 4.7% of pairs of SNPs in uLD), despite genetic distances in the *CYP4F* cluster being four times larger than in the *CYP3A* cluster (maximum distance of 0.60 cM vs 0.15 cM, respectively), whereas the physical size of the cluster is only twice (500 Kb vs 250 Kb, respectively). Significant uLD between *CYP3A5* and *CYP3A43* and between *CYP3A7* and *CYP3A43* can be seen in all European populations (supplementary fig. S3a, Supplementary Material online). *CYP3A5* and *CYP3A43* are the opposite to each other in term of physical location in the cluster while *CYP3A7* and *CYP3A43* are next to each other. Finland (FIN) and Toscani (TSI) populations have the most uLD signals across European populations, with FIN uniquely showing uLD between *CYP3A5*-*CYP3A4*, and TSI showing uLD between *CYP3A4* and *CYP3A43*, a signal consistently seen in the East Asians. TSI also have the highest genetic distance interval in this region, likely due to a larger, more widespread, recombination rate in *CYP3A4* compared to other populations (supplementary fig. S4, Supplementary Material online). Among East Asians, uLD signals are seen almost exclusively between SNPs in *CYP3A4* and *CYP3A43*, two genes that are next to each other, with no clear recombination hotspot separating them, meaning that linkage disequilibrium can be expected (supplementary fig. S4, Supplementary Material online). SNPs in these genes are also in uLD in Gujarati Indian (GIH) population, but none of the other South Asian populations show any signal, which may be explained by the short genetic distances within this cluster in this super-population (SAS) (<0.05 cM). Finally, African populations show the most deviation from the null (Fig. 4a). SNPs in *CYP3A4* are in uLD with all other genes and the signal also replicates the observations from the European populations, with SNPs in *CYP3A43* in uLD with SNPs in all other genes (Fig. 4a, supplementary fig. S3a, Supplementary Material online).

In the *CYP4F* cluster, several pairs of SNPs have patterns of LD that deviate significantly from the empirical distribution (supplementary fig. S3b, Supplementary Material online). There is uLD for *CYP4F22*-*CYP4F11* and *CYP4F22*-*CYP4F12* in almost every populations, even though these genes are far from each other (0.36 and 0.12 Mb, respectively). *CYP4F22* and *CYP4F2* are also in uLD in AFR, EUR, and EAS.

The African populations have more evidence of uLD than the other super-populations. One population in particular, the Yoruba (YRI) population, has even more extreme signals in comparison with other African populations and most uLD signal are driven by associations involving the *CYP4F12* gene (Fig. 4b). Thus, we investigated whether a specific region in *CYP4F12* is in strong LD with the other genes. Indeed, in the YRI population, there is evidence of uLD between a region in *CYP4F12* (at 15.79–18.00 Mb on chromosome 19) and the *CYP4F3* (supplementary fig. S5a, Supplementary Material online) and *CYP4F8* genes (supplementary fig. S5b, Supplementary Material online). The extreme signals in this gene cluster are in line with the hypothesis that balancing selection acts via gene–gene interactions, or epistasis (Llaurens et al. 2017). As these patterns could be due to sequencing errors (Akey et al. 2001), we used the latest 1000G dataset which has high-coverage sequencing and is aligned on GRCh38 (Materials and Methods). These results were replicated in this second dataset, greatly reducing the possibility that the observed signal is due to sequencing errors or spurious mapping. Finally, in the Europeans, the FIN population has a specific pattern between *CYP4F12* and *CYP4F2*, *CYP4F8*, and *CYP4F3*. Looking more closely, many SNPs in *CYP4F12* are in uLD with one SNP in *CYP4F3* (supplementary fig. S5a, Supplementary Material online) and two SNPs in *CYP4F8* (supplementary fig. S5b, Supplementary Material online). No specific SNPs are in uLD with *CYP4F2*.

### Detection of eQTLs

We next evaluated the effects of the SNPs identified as being under positive and balancing selection on the expression of the genes in each *CYP450* cluster to test if these are eQTLs.

**Fig. 5.**
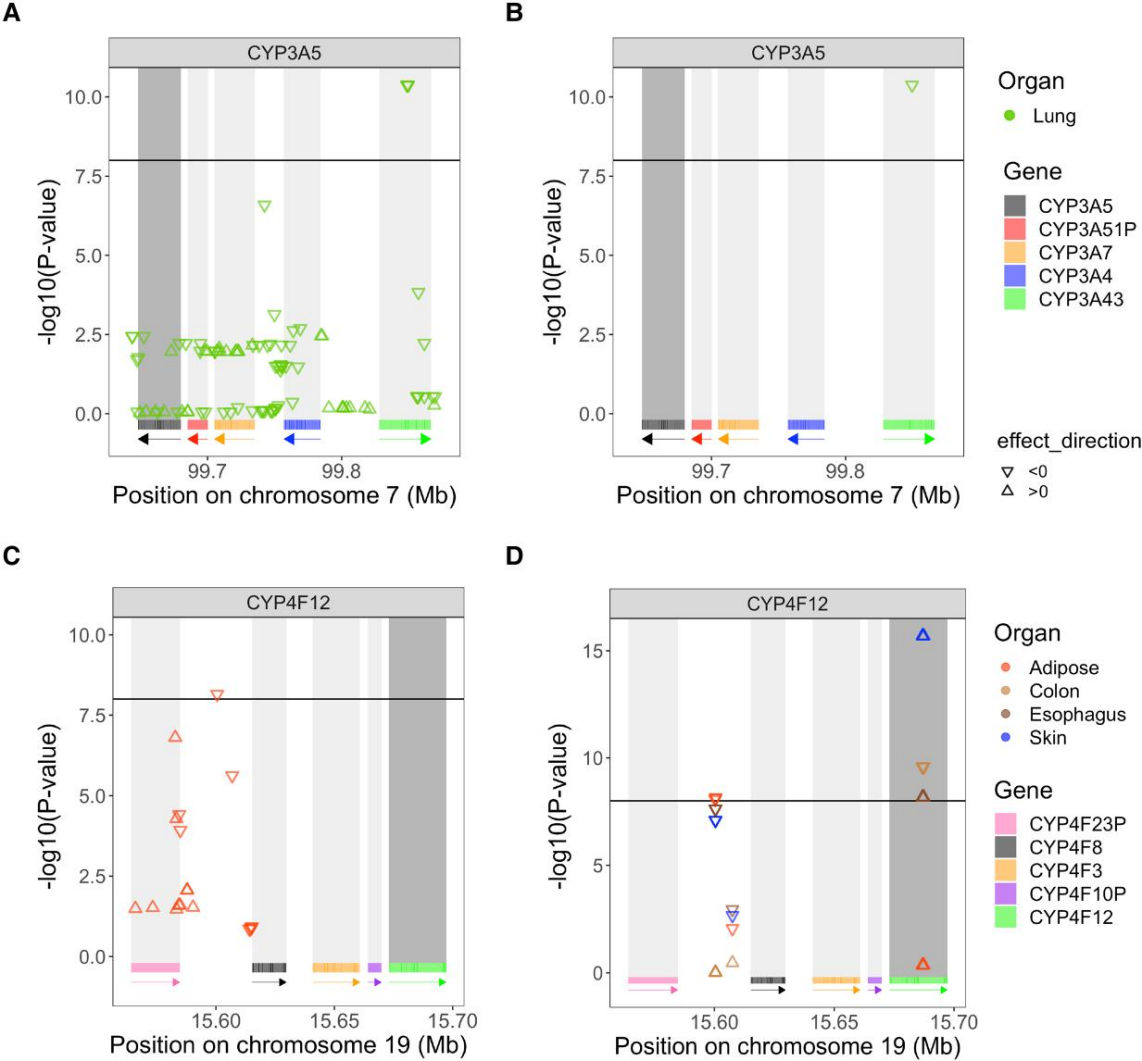
*P*-values of the associations between SNPs under a) positive selection and b) balancing selection and CYP3A5’s gene expression in lung and *P*-values associated with SNPs c) under positive selection and d) balancing selection and tissue-specific gene expression of CYP4F12. CYP3A5 and CYP4F12 are shown in dark gray, as the expressions of these genes are tested. The triangle standing on its base indicates a positive effect size ($\beta_{eQTL}>0$), while a triangle standing on its point indicates a negative effect size ($\beta_{eQTL}<0$). The threshold, set to $10^{-8}$, is represented by the horizontal black line, meaning that a −log10(P−value)>8 is a significant eQTL. Only tissues with significant eQTLS are displayed. As before, rectangles below each plot show the position of each gene, and arrows indicate on which strand the gene is located. Each gene has its own color to indicate its location.

In the *CYP3A* cluster, three SNPs are under positive selection in the Punjabi population from South Asia (PJL): rs487813, rs679320, and rs568859. These SNPs are located in *CYP3A43* and are significant eQTLs of *CYP3A5* in lung (Fig. 5a). The SNP under balancing selection in the Luhya population (LWK) in *CYP3A43*, rs800667, is also an eQTL of *CYP3A5* in lung (Fig. 5b). The effect size estimate for these significant eQTL is negative, indicating a reduction in *CYP3A5* gene expression with each nonreference allele. This locus in *CYP3A43* thus impact *CYP3A5* expression in lung, even though *CYP3A5* and *CYP3A43* are at opposite ends of the cluster, 147.99 Kb apart. According to the ReMap density database (Hammal et al. 2022), this locus also displays regulatory signals, supporting the importance of this region at the transcriptional regulatory level. This result is in line with the LD analyses (Fig. 4a), which suggested uLD between SNPs in *CYP3A5* and *CYP3A43* in Europeans, Africans, and the Japanese. Those four SNPs were all in uLD with 11 SNPs in the Toscani population (TSI) and five other SNPs in Americans of African Ancestry (ASW).

In the *CYP4F* cluster, a SNP under positive selection, rs74459786 (supplementary table S1, Supplementary Material online), located in the intergenic region between *CYP4F23P* and *CYP4F8*, is an eQTL of *CYP4F12* in adipose tissue (Fig. 5c), with a negative effect size. SNPs under balancing selection (supplementary table S2, Supplementary Material online) within *CYP4F12* are eQTLs for *CYP4F12* expression in the colon, esophagus, and skin, but interestingly, their effects in these tissues are in opposite directions, with positive effect sizes in the colon and skin, and negative ones for the esophagus. Furthermore, a SNP with a balancing selection signal is also an eQTL of *CYP4F12* expression in adipose-subcutaneous tissue (Fig. 5d) with a negative effect size estimate. It lies in the intergenic region between *CYP4F23P* and *CYP4F8*, which is the same region as the SNP under positive selection (rs74459786) in Fig. 5c.

Another SNP under positive selection in this intergenic region, rs62115147 (supplementary table S1, Supplementary Material online), is also associated with *CYP4F3* expression in one of the brain tissues (Brain-Spinalcord-cervicalc-1) and in nerve tissue (supplementary fig. S6a, Supplementary Material online). The *CYP4F12* gene emerged repeatedly as a candidate in our balancing selection and uLD analyses, while the intergenic region between *CYP4F23P* and *CYP4F8* is seen only in the balancing selection analysis.

Even if less positive selection is present in the *CYP4F* cluster compared to the *CYP3A* cluster, many of the SNPs showing high iHS values in the *CYP4F* cluster show up as eQTLs for different genes. SNPs under positive selection located in *CYP4F11* (supplementary table S1, Supplementary Material online) are eQTLs of *CYP4F2* in brain and skin tissues (supplementary fig. S6b, Supplementary Material online) with consistent, negative effect sizes. Additionally, the same SNPs under positive selection within *CYP4F11* are associated with expression of *CYP4F11* itself in multiple tissues (supplementary fig. S6c, Supplementary Material online). The direction of effect on gene expression is the same for all significant associations.

### Phenotypic Associations

Using the UK Biobank cohort (UKb), we did a Phenome-Wide Association Study (PheWAS) to identify phenotypes potentially under selective pressure (Materials and Methods), using the available variants with selective signals in the *CYP4F* genes (166 variants from the 180 found under selection) and in the *CYP3A* genes (62 from the 125 variants found under selection). No significant associations were found for SNPs under selective pressure in *CYP4F* cluster.

**Fig. 6.**
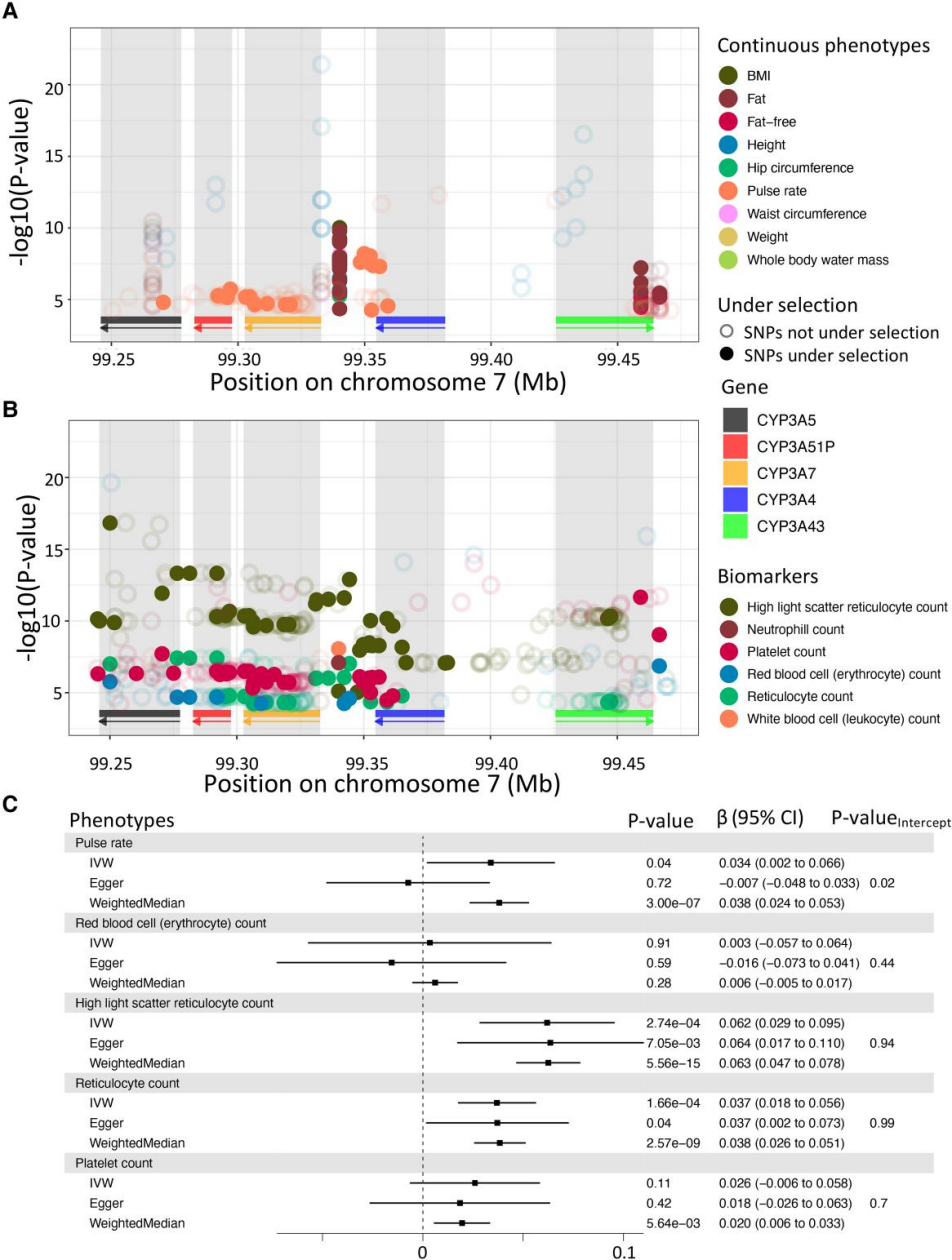
Associations of CYP3A cluster with phenotypes in the UK biobank. Significant associations (P<0.05/777) for continuous traits a) and plasmatic biomarkers b) which are significant in at least one SNP under selection. SNPs under selection are represented as full dots, meanwhile other SNPs are represented as empty dots. As before, rectangles below each plot show the position of each gene, and arrows indicate on which strand the gene is located. Each gene has its own color to indicate its location. c) Causal relationship with CYP3A5 expression in lung for phenotypes showing significant association with its eQTLs. *β* represents the change of 1 standard deviation of CYP3A5 expressions on phenotypes, also in standard deviation units. *P*-value of three statistics (IVW, Egger, Weighted Median) are displayed with the *β* and the 95% confidence interval (CI) of the association for each phenotype in the gray box. For Egger, the *P*-value of the intercept is also displayed.

In the CYP3A cluster, however, SNPs under positive selection in at least one studied populations were found associated with six phenotypes (Fig. 6a,b) in our PheWAS. Among the disease phenotypes, we found association with pelvic inflammatory disease (PID), which is female-specific, and for which the SNP with the strongest association ($P{-\mathrm{value}}_{rs2014764}=1.96\times 10^{-5}$) was under positive selection in European (CEU, GBR) and East Asian (CHB, CHS, CDX) populations. Among the continuous phenotypes investigated (Materials and Methods), we found association with pulse rate, for which the SNP with the strongest association ($P{-\mathrm{value}}_{rs12536946}=4.66\times 10^{-13}$) was also found under selective pressure in Europeans (CEU). Among the biomarker variables, the strongest associations with platelet count ($P{-\mathrm{value}}_{rs503115}=2.30\times 10^{-12}$) and erythrocyte count ($P{-\mathrm{value}}_{rs10235630}=1.83\times 10^{-7}$) were both found with SNPs under selective pressure in the Japanese population.

Lastly, for both high light scatter reticulocyte count and reticulocyte count, their strongest association ($P{-\mathrm{value}}_{rs73713580}=1.24\times 10^{-17}$; $P{-\mathrm{value}}_{rs55830753}=3.08\times 10^{-8}$ respectively) were both found under selective pressure in African population (MSL and ACB, respectively). Using Mendelian randomization (Materials and Methods), we evaluated the causal relationship between *CYP3A5* expression in lung, for which eQTLs were found under selective pressure above, and the phenotypes found to be associated with SNPs in the *CYP3A* cluster. We identified a significant causal association between CYP3A5 expression and both high light scatter reticulocyte count ($P{-\mathrm{value}}_{\mathrm{IVW}}=2.74\times 10^{-4}$) and reticulocyte count ($P{-\mathrm{value}}_{\mathrm{IVW}}=1.66\times 10^{-4}$). We did not detect pleiotropy using MR-Egger and results were robust using the weighted median test (Materials and Methods).

Altogether, these results indicate that the selective pressure in the *CYP3A* cluster could be driven by the production of reticulocyte through the expression levels of *CYP3A5* and also suggest that pulse rate could be impacted by genetic variation in *CYP3A* genes.

Among other associations identified in this cluster, three SNPs showed strong associations with anthropometric traits (Fig. 6a) and are under selective pressure in European population (CEU, IBS). Those SNPs were, however, found to be associated with expression of genes outside the *CYP3A* cluster (supplementary text 14.3, Supplementary Material online), prompting for further investigation of the relationship between this cluster and other neighboring genes to understand the different drivers at play.

## Discussion

Drug metabolism is a rather complex system with the *CYP450* genes metabolizing around 75% of common drugs. As shown by others (Thompson et al. 2004; Qiu et al. 2008; Chen et al. 2009; Li et al. 2011), we also found that selective pressure and genetic differentiation between populations were present in *CYP450* genes. Here, we provide a deeper analysis of two *CYP450* clusters, the widely studied CYP3A (Burk and Wojnowski 2004; Thompson et al. 2004, 2006; Chen et al. 2009) and the less well-known *CYP4F* clusters, identified thanks to their outlier patterns in neutrality and population differentiation analyses. These two *CYP450* clusters exhibit multiple selective signatures (positive selection and balancing selection) and show population differentiation. We found that natural selection forces involved differ between the two clusters; the *CYP3A* cluster is evolving under positive selection, while the *CYP4F* cluster show signals of balancing selection. Furthermore, the *CYP4F* cluster shows strong evidence for co-evolution and co-regulation signals.

In the literature, the *CYP450* genes are often studied independently. In our study, we considered the evolution of the entire family cluster, mostly *CYP3A* and *CYP4F* genes, and detected signatures of co-evolution between the paralogous genes, suggestive of potential epistatic interactions. As these clusters of genes are involved in drug metabolism (Danielson 2002; Nebert and Dalton 2006; Liang et al. 2012; Zhang et al. 2017), it is important to understand the impact of genetic variants on their gene expression, to help understand how these variants might impact drug response and refine disease treatments in a personalized way. Our results also show that the impact of specific variants may differ between populations, which could lead to a deeper understanding of differences in individual drug response (Singh et al. 2011; Guttman et al. 2019).

The *CYP3A* cluster contains four genes: *CYP3A4*, *CYP3A5*, *CYP3A7*, and *CYP3A43*. Signals of positive selection were detected in the *CYP3A* cluster, specifically in *CYP3A4* and *CYP3A7*, which have been under recent positive selection in African, European and the Chinese populations, while *CYP3A5* appears under positive selection in Europeans and *CYP3A43* in non-Africans (Chen et al. 2009). Our analyses confirmed that *CYP3A* genes are evolving under positive selection as previously reported (Thompson et al. 2004; Voight et al. 2006; Qiu et al. 2008; Li et al. 2011).

We found that the locus known to cause nonexpression of *CYP3A5* (Kuehl et al. 2001), rs10264272/CYP3A5*6, is under positive selection (|iHS|≥2) in African populations (YRI, GWD, LWK). A second locus, known to cause low *CYP3A5* expression, rs776746/CYP3A5*3, is under positive selection (|iHS|≥2) in two African populations (YRI, GWD). These derived allele have thus swept up in frequency in several African populations. In the Toscani population, rs776746/CYP3A5*3 is found to be in uLD with the four SNPs under selective pressure in the *CYP3A* cluster, that are eQTLs of *CYP3A5* in lung.

*CYP3A43* is the ancestor gene of this cluster (McArthur et al. 2003; Qiu et al. 2008); however, its function is not well understood, unlike other *CYP3A* genes. Our analyses suggest that SNPs in *CYP3A43* regulate *CYP3A5* gene expression, at least in lung. Levels of expression of *CYP3A5* in lung were causally associated to reticylocytes count and many of its eQTLs were under selection in Africans. Since *Plasmodium vivax*, a parasite causing malaria, affect mainly young reticulocytes (Clark et al. 2021) and that malaria is present in Africa, the selective pressure found in this population could be associated to this disease. Further studies need to be done to validate this hypothesis.

In the *CYP4F* cluster, we found both positive and balancing selection pressures acting. Furthermore, the SNPs evolving under selective pressures are associated with gene expression levels across the cluster in several tissues. For instance, a cis-eQTL of *CYP4F12*, rs74459786, is detected to be under positive selection in the Kinh population in East Asia (KHV). We also found that several SNPs in *CYP4F11* are associated with *CYP4F2* expression. Both genes are implicated in common metabolic function, such as the synthesis of 20-hydroxyeicosatetraenoic acid (20-HETE) from arachidonic acid (Yi et al. 2017). Thus, this could indicate a possible regulatory mechanism of common functions.

Finally, the region between *CYP4F23P* and *CYP4F8* emerged multiple times in our analyses. This intergenic region shows strong signals for selection, with the same SNPs also being eQTLs of *CYP4F3* (nerve) and *CYP4F12* (adipose tissue). Given the implication of *CYP4F12* in fatty acid metabolism (Stark et al. 2005), our results may point towards the identification of new regulatory elements involved in this process in adipose tissues.

A potential limitation in the current study is that population genetic statistics can be biased in the presence of fine-scale population structure. However, to mitigate this issue, we performed our analyses not only at the broader population level but also within individual subpopulations, ensuring that the values obtained from subpopulations were consistent with those from the overall superpopulation.

An important limitation to consider is the methodology used for calculating Tajima’s D using the vcftools software. This tool’s current implementation does not account for mappability and callability in whole genome sequencing data. This approach introduces a bias by implicitly considering uncalled positions as nonvariable, leading to an underestimation of diversity measures (Korunes and Samuk 2021). Although our strategy to exclude regions with high proportions of missing data likely minimizes this bias, we acknowledge that approaches that directly incorporate genomic accessibility considerations for a more precise estimation of genetic diversity should be used in future studies of CYP genes. While the impact on our results appears minimal, as evidenced by the high similarity of results after the removal of high-missing data regions, the potential for underestimation of diversity estimators should be considered when comparing these estimates across genomic regions.

In conclusion, our results demonstrate high heterogeneity across human populations, both in terms of selective signals and interaction between variants and expression levels, for the *CYP3A* and *CYP4F* genes. There could thus be important differences in metabolic regulation impacting drug response in individuals from different ethnicities. In particular, these variants could cause impaired efficacy, as well as side effects. As pharmacogenetic studies still typically focus on European populations, our results underline the importance of including individuals from several populations in order to capture all of the genetic diversity and its impact on disease treatment and metabolism.

## Materials and Methods

### 1000 Genomes Genetic Data

The data analyzed are from the phase III of the 1000 Genomes project (1000G) (Genomes Project Consortium et al. 2015). The 1000 Genomes Project includes 2,504 individuals from 26 populations. These populations can be split into five distinct genetic ancestries, referred herein as super-populations, as defined by the 1000G consortium: African (AFR), European (EUR), South Asian (SAS), East Asian (EAS), and Admixed American (AMR). Data from the AMR population are not included in this study because the high degree of admixture may confound selection and linkage disequilibrium analyses. This left us with 22 subpopulations and four super-populations for study. The available variant call format (vcf) files of 1000G are under the GRCh37 genome build. VCFtools v0.1.14 (Danecek et al. 2011) was used to filter the 1000G dataset. Indels and nonbiallelic alleles were removed and only SNPs located in the 57 CYP450 genes were kept, extracted based on coordinates genomic coordinates obtained from the UCSC genes table using the UCSC Genome Browser (supplementary file 1, Supplementary Material online). After filtering, the CYP450 dataset included a total of 61,739 SNPs and 2,157 individuals. We refer to this as the “1000G CYP450 dataset.” A more recent dataset was also used as a validation dataset for the unusual linkage disequilibrium analysis, the resequencing dataset of 30X coverage, mapped on GRCh38 (Byrska-Bishop et al. 2021), which includes the 2157 individuals.

### Genetic Diversity and Population Differentiation

Both Tajima’s D and $F_{ST}$ statistics were obtained with VCFtools (Danecek et al. 2011) using the 1000G CYP450 dataset (Genomes Project Consortium et al. 2015). Tajima’s D values were calculated in the super-population (AFR, EUR, EAS, and SAS) separately on nonoverlapping windows of 1 Kb. We also performed these analyses excluding positions and windows with low mappability (see supplementary text 14.1 and fig. S2, Supplementary Material online). We computed the mean Tajima’s D value for each gene by averaging the window-based values, and sorted genes according to their mean. To create a null distribution, we computed Tajima’s D values for all SNPs associated with a gene name in the Combined Annotation Dependent Depletion (CADD) annotation file (Kircher et al. 2014) on chromosome 22, so that all SNPs used to compute the empirical distribution are located in genes. We computed the 2.5 and 97.5th percentile on the window-based values of chromosome 22. Values above the 97.5th percentile and below the 2.5th percentile were considered to be statistically significant (two sided empirical P−value<0.05). To ensure that our results were not biased by fine-scale population structure, we also perform the analyses in each of the subpopulations of EUR (see supplementary text 14.1 and fig. S2, Supplementary Material online). The $F_{ST}$ values, from Weir and Cockerham derivation (Weir and Cockerham 1984), were calculated using four super-populations (AFR, EUR, EAS, and SAS) on a per-site basis. The per-gene mean was calculated on raw values and genes were sorted based on their mean $F_{ST}$. As in the previous analysis, chromosome 22 was used to create an empirical distribution. $F_{ST}$ values were also computed on SNPs located in genes of the chromosome 22 (see above) and the per-gene mean $F_{ST}$ was calculated.

### Detecting Natural Selection

The method used to detect balancing selection is the *β* score (Siewert and Voight 2017). This score has already been calculated on the whole 1000 Genomes project data for each subpopulation. The approach used to detect signal of recent positive selection was iHS (integrated haplotype score) (Voight et al. 2006). The iHS computation was performed by us on the 1000G dataset, filtered to exclude INDELs and CNVs. Reference alleles from filtered 1000 Genomes vcf files were changed to the ancestral alleles retrieved from six primates EPO pipeline (version e59) using the fixref plugin of bcftools (Li 2011). The hapbin program v.1.3.0 (Maclean et al. 2015) was then used to compute iHS using per population-specific genetic maps computed by Adam Auton on the 1000 Genomes OMNI dataset (Genomes Project Consortium et al. 2015). When the genetic map was not available for a subpopulation, the genetic map from the closest subpopulation was selected according to their global $F_{ST}$ value computed on the 1000G dataset. For all natural selection analyses, SNPs annotated to be in a repetitive region were identified using the RepeatMasker track available on the UCSC genome browser (Kent et al. 2002) and were removed.

### Unusual Linkage Disequilibrium

Linkage disequilibrium between pairs of SNPs from the same cluster was assessed using the geno-r2 option from VCFTools on SNPs with minor allele frequencies (MAF) above 0.05. The genetic position of each SNP was calculated with PLINK v1.90 (Chang et al. 2015) using the population-specific genetic maps the same was as described in previous section.

To compute a null distribution to detect unusual linkage disequilibrium (uLD), the Human GRCh38 Gene transfer format (GTF) file from Ensembl v87 was screened per autosomal chromosome using an in-house python script to find windows matching the *CYP4F* cluster: windows of 430 Kb containing six genes were kept. In these windows, we excluded INDELs and SNPs with MAF <0.05. The $r^{2}$ for each pair of SNPs located within a selected window was computed using VCFTools with the geno-r2 option. We divided the genetic distance into bins of 0.01 cM and calculated the 99th percentile of $r^{2}$ values of each pair of SNPs lying in the bin. This process was done separately for each 1000G subpopulation, yielding a null distribution per subpopulation. $r^{2}$ values on pairs of SNPs in the extremes of the empirical distribution are considered to be significant for what we called unusual linkage disequilibrium (uLD).

To specifically confirm the signal seen between *CYP4F12* and other *CYP4F* genes, we extracted only the SNPs showing significant uLD in the previous analysis and kept only those pairs where one SNP was located in *CYP4F12*. Using VCFTools, *CYP4F* genetic data were extracted from the 1000 Genomes 30X on GRCh38 dataset (Byrska-Bishop et al. 2021), and $r^{2}$ values were calculated as described above.

### eQTLs Analysis of SNPs under Selection

The Genotype-Tissue Expression v8 (GTEx) (Lonsdale et al. 2013) was accessed through dbGaP (phs000424.v8.p2, dbgap project #19088) and contains gene expression across 54 tissues and 948 donors as well as genotyping information, compiled in a VCF file by GTEx on the GRCh38 genome build. The cohort comprises 67% males and 33% females, mainly of European descent (84.6%), aged between 20 and 79 years old. Analyses were done on 699 individuals of European descent, as described in supplementary text 14.2, Supplementary Material online. To take into account hidden factors, we calculated PEER factors on the normalized expressions. We removed tissues with less than 50 samples, leaving samples from 50 different tissues.

For eQTL analyses, we selected only SNPs that were identified to be under positive or balancing selection in CYP3A and CYP4F clusters in previous analyses and with a MAF above 5%. Since the positions of these SNPs were in the GRCh37 genome build, we converted these positions to the GRCh38 genome build to match GTEx v8 data, using the liftOver function of the rtracklayer R library (Lawrence et al. 2009). *P*-values of associations between each selected SNP and gene expression of every gene in the cluster were calculated with a linear model using the lm function in R. The linear model was calculated on each SNP individually. The covariates include the first five principal components (PCs) (see supplementary text 14.2, Supplementary Material online), age, sex, PEER factors, the collection site (SMCENTER), the sequencing platform (SMGEBTCHT), and total ischemic time (TRISCHD). To report genome-wide significant eQTL signals, we used a *P*-value threshold for significance at $10^{-8}$.

Lastly, we have searched for regulatory annotations of at eQTL signals using the UCSC Genome Browser, specifically looking at the data provided by the ReMap density database (Hammal et al. 2022).

### Phenotypic Associations

The UK biobank (UKb) (Sudlow et al. 2015) was accessed through project 15357. We kept only individuals of European descent which were within 3 SD of the mean for the top 3 PCs, removed one individual for each pair of related individuals, and removed individuals whose genetic sex did not match self-identified sex. We extracted positions for CYP3A (chr7:99-244-812-99-470-881, GRCh38) and for CYP4F (chr19:15-618-335-16-110-830, GRCh38) families. We then removed positions with more than 10% of missing genotype and with a MAF under 1%, then removed individuals with more than 5% missing genotypes, leaving us with 399,149 individuals and 3,092 variants for the CYP4F genes, and 400,504 individuals and 374 variants for the CYP3A genes.

We used baseline values for continuous phenotypes. We selected phenotypes recommended by the UKb, as well as blood cells measurements, a total of 90 and 11 phenotypes respectively (supplementary table S3, Supplementary Material online). When many values were available at the baseline, we took the mean of those values. We also looked at diseases using phecode coding extracted from phewascatalog (Denny et al. 2013), which indicated ICD-10 to group and to exclude from controls. We kept only phecodes with more than 500 cases, leaving 603 for both sexes, 62 female-only and 11 male-only. Covariates used are the age at baseline, sex, top 10 PCs, deprivation index and the genotyping array. Analyses were done with plink2 (Chang et al. 2015) with linear transformation of the quantitative covariates. We used a *P*-value threshold for significance at $6.44\times 10^{-5}$, based on Bonferonni correction for the number of phenotypes evaluated ($0.05/(90+11+603+62+11)=6.44\times 10^{-5}$).

We performed Mendelian randomization analyses. As instrument variables, we selected SNPs in *CYP3A* cluster showing strong associations with *CYP3A5* expression in lung (exposure), with a F-statistic above 10 and a *P*-value under 0.001 (0.05/50 tissues), then removed SNPs in pair with a correlation above $r^{2}>0.8$, estimated on Europeans from GTEx using the function ld_matrix from ieugwasr package in R (Lyon et al. 2021), leaving eight SNPs for analyses. Furthermore, we used the scale function on continuous traits and gene expression to estimate the change of 1 SD of the phenotypes for 1 SD of the gene expression. As outcomes, we used the six phenotypes for which SNPs under selection showed associations for both phenotype ($P-\mathrm{value}<6.44\times 10^{-5}$) and CYP3A5 expression (P−value<0.001). Mendelian randomization analyses were performed using MendelianRandomization package in R (Yavorska and Burgess 2017) and correlation matrix generated using ld_matrix (Lyon et al. 2021) was given to the function to adjust for linkage disequilibrium. We performed Inverse Variance Weighted (IVW) as the main statistical test, and we performed MR-Egger to detect and correct for directional pleiotropy: we report MR-Egger results if the intercept was significant. Lastly, we performed weighted median test as a sensitivity test.

## Supplementary Material

evad236_Supplementary_Data

## Data Availability

The 1000 Genomes Project, GEUVADIS is freely available. The GTEx v8 dataset was accessed through dbGaP under project number #19088. The UK Biobank was accessed through data access approval under the project number #15357. Information to apply for data access can be found here: https://www.ukbiobank.ac.uk/enable-your-research/apply-for-access.

## References

[evad236-B1] Akey JM , Zhang K, Xiong M, Doris P, Jin L. The effect that genotyping errors have on the robustness of common linkage-disequilibrium measures. Am J Hum Genet. 2001:68(6):1447–1456. 11359212 10.1086/320607 PMC1226131

[evad236-B2] Aqil A , Speidel L, Pavlidis P, Gokcumen O. Balancing selection on genomic deletion polymorphisms in humans. eLife. 2023:12:e79111. 36625544 10.7554/eLife.79111 PMC9943071

[evad236-B3] Bains RK , Kovacevic M, Plaster CA, Tarekegn A, Bekele E, Bradman NN, Thomas MG. Molecular diversity and population structure at the Cytochrome P450 3A5 gene in Africa. BMC Genet. 2013:14:34–34. 23641907 10.1186/1471-2156-14-34 PMC3655848

[evad236-B4] Brown S-A , Pereira N. Pharmacogenomic impact of CYP2C19 variation on clopidogrel therapy in precision cardiovascular medicine. J Pers Med. 2018:8(1):8. 29385765 10.3390/jpm8010008 PMC5872082

[evad236-B5] Burk O , Wojnowski L. Cytochrome P450 3A and their regulation. Naunyn Schmiedebergs Arch Pharmacol. 2004:369(1):105–124. 14569421 10.1007/s00210-003-0815-3

[evad236-B6] Byrska-Bishop M , Evani US, Zhao X, Basile AO, Abel HJ, Regier AA, Corvelo A, Clarke WE, Musunuri R, Nagulapalli K, et al. High coverage whole genome sequencing of the expanded 1000 Genomes Project cohort including 602 trios. bioRxiv 430068. 10.1101/2021.02.06.430068, 2021. PMC9439720 36055201

[evad236-B7] Carlson CS , Thomas DJ, Eberle MA, Swanson JE, Livingston RJ, Rieder MJ, Nickerson DA. Genomic regions exhibiting positive selection identified from dense genotype data. Genome Res. 2005:15(11):1553–1565. 16251465 10.1101/gr.4326505 PMC1310643

[evad236-B8] Chang CC , Chow CC, Tellier LC, Vattikuti S, Purcell SM, Lee JJ. Second-generation plink: rising to the challenge of larger and richer datasets. Gigascience. 2015:4:7. 25722852 10.1186/s13742-015-0047-8 PMC4342193

[evad236-B9] Chen X , Wang H, Zhou G, Zhang X, Dong X, Zhi L, Jin L, He F. Molecular population genetics of human CYP3A locus: signatures of positive selection and implications for evolutionary environmental medicine. Environ Health Perspect. 2009:117(10):1541–1548. 20019904 10.1289/ehp.0800528 PMC2790508

[evad236-B10] Clark MA , Kanjee U, Rangel GW, Chery L, Mascarenhas A, Gomes E, Rathod PK, Brugnara C, Ferreira MU, Duraisingh MT. Plasmodium vivax infection compromises reticulocyte stability. Nat Commun. 2021:12(1):1629. 33712609 10.1038/s41467-021-21886-x PMC7955053

[evad236-B11] Danecek P , Auton A, Abecasis G, Albers CA, Banks E, DePristo MA, Handsaker RE, Lunter G, Marth GT, Sherry ST, et al. The variant call format and VCFtools. Bioinformatics. 2011:27(15):2156–2158. 21653522 10.1093/bioinformatics/btr330 PMC3137218

[evad236-B12] Danielson PB . The cytochrome P450 superfamily: biochemistry, evolution and drug metabolism in humans. Curr Drug Metab. 2002:3(6):561–597. 12369887 10.2174/1389200023337054

[evad236-B13] Denny JC , Bastarache L, Ritchie MD, Carroll RJ, Zink R, Mosley JD, Field JR, Pulley JM, Ramirez AH, Bowton E, et al. Systematic comparison of phenome-wide association study of electronic medical record data and genome-wide association study data. Nat Biotechnol. 2013:31(12):1102–1111. 24270849 10.1038/nbt.2749 PMC3969265

[evad236-B14] Elens L , van Gelder T, Hesselink DA, Haufroid V, van Schaik RH. CYP3A4*22: promising newly identified CYP3A4 variant allele for personalizing pharmacotherapy. Pharmacogenomics. 2012:14(1):47–62. 10.2217/pgs.12.187 23252948

[evad236-B15] Gaedigk A . Complexities of CYP2D6 gene analysis and interpretation. Int Rev Psychiatry. 2013:25(5):534–553. 24151800 10.3109/09540261.2013.825581

[evad236-B16] Gaedigk A , Sangkuhl K, Whirl-Carrillo M, Klein T, Leeder J S. Prediction of CYP2D6 phenotype from genotype across world populations. Genet Med. 2017:19(1):69–76. 27388693 10.1038/gim.2016.80 PMC5292679

[evad236-B17] Genomes Project Consortium , et al. A global reference for human genetic variation. Nature. 2015:526(7571):68–74. 26432245 10.1038/nature15393 PMC4750478

[evad236-B18] Guttman Y , Nudel A, Kerem Z. Polymorphism in cytochrome P450 3A4 is ethnicity related. Front Genet. 2019:10:224. 30941162 10.3389/fgene.2019.00224 PMC6433705

[evad236-B19] Hammal F , de Langen P, Bergon A, Lopez F, Ballester B. Remap 2022: a database of human, mouse, drosophila and arabidopsis regulatory regions from an integrative analysis of DNA-binding sequencing experiments. Nucleic Acids Res. 2022:50(D1):D316–D325. 34751401 10.1093/nar/gkab996 PMC8728178

[evad236-B20] Huang Y , Wuchty S, Przytycka TM. eQTL epistasis – challenges and computational approaches. Front Genet. 2013:4:51. 23755066 10.3389/fgene.2013.00051 PMC3668133

[evad236-B21] Janha RE , Worwui A, Linton KJ, Shaheen SO, Sisay-Joof F, Walton RT. Inactive alleles of cytochrome P450 2C19 may be positively selected in human evolution. BMC Evol Biol. 2014:14:71. 24690327 10.1186/1471-2148-14-71 PMC4036532

[evad236-B22] Kent WJ , Sugnet CW, Furey TS, Roskin KM, Pringle TH, Zahler AM, Haussler D. The human genome browser at UCSC. Genome Res. 2002:12(6):996–1006. 12045153 10.1101/gr.229102 PMC186604

[evad236-B23] Kim Y , Stephan W. Detecting a local signature of genetic hitchhiking along a recombining chromosome. Genetics. 2002:160(2):765–777. 11861577 10.1093/genetics/160.2.765 PMC1461968

[evad236-B24] Kircher M , Witten DM, Jain P, O’Roak BJ, Cooper GM, Shendure J. A general framework for estimating the relative pathogenicity of human genetic variants. Nat Genet. 2014:46(3):310–315. 24487276 10.1038/ng.2892 PMC3992975

[evad236-B25] Kirischian NL , Wilson JY. Phylogenetic and functional analyses of the cytochrome P450 family 4. Mol Phylogenet Evol. 2012:62(1):458–471. 22079551 10.1016/j.ympev.2011.10.016

[evad236-B26] Korunes KL , Samuk K. pixy: unbiased estimation of nucleotide diversity and divergence in the presence of missing data. Mol Ecol Resour. 2021:21(4):1359–1368. 33453139 10.1111/1755-0998.13326 PMC8044049

[evad236-B27] Kudaravalli S , Veyrieras J-B, Stranger BE, Dermitzakis ET, Pritchard JK. Gene expression levels are a target of recent natural selection in the human genome. Mol Biol Evol. 2009:26(3):649–658. 19091723 10.1093/molbev/msn289 PMC2767089

[evad236-B28] Kuehl P , Zhang J, Lin Y, Lamba J, Assem M, Schuetz J, Watkins PB, Daly A, Wrighton SA, Hall SD, et al. Sequence diversity in CYP3A promoters and characterization of the genetic basis of polymorphic CYP3A5 expression. Nat Genet. 2001:27(4):383–391. 11279519 10.1038/86882

[evad236-B29] Lamba J , Hebert JM, Schuetz EG, Klein TE, Altman RB. PharmGKB summary: very important pharmacogene information for CYP3A5. Pharmacogenet Genomics. 2012:22(7):555–558. 22407409 10.1097/FPC.0b013e328351d47f PMC3738061

[evad236-B30] Lawrence M , Gentleman R, Carey V. rtracklayer: an R package for interfacing with genome browsers. Bioinformatics. 2009:25(14):1841–1842. 19468054 10.1093/bioinformatics/btp328 PMC2705236

[evad236-B31] Lehner B . Molecular mechanisms of epistasis within and between genes. Trends Genet. 2011:27(8):323–331. 21684621 10.1016/j.tig.2011.05.007

[evad236-B32] Li H . A statistical framework for SNP calling, mutation discovery, association mapping and population genetical parameter estimation from sequencing data. Bioinformatics. 2011:27(21):2987–2993. 21903627 10.1093/bioinformatics/btr509 PMC3198575

[evad236-B33] Li J , Zhang L, Zhou H, Stoneking M, Tang K. Global patterns of genetic diversity and signals of natural selection for human ADME genes. Hum Mol Genet. 2011:20(3):528–540. 21081654 10.1093/hmg/ddq498

[evad236-B34] Liang R , Wang C, Zhao H, Huang J, Hu D, Sun Y. Influence of CYP4F2 genotype on warfarin dose requirement—a systematic review and meta-analysis. Thromb Res. 2012:130(1):38–44. 22192158 10.1016/j.thromres.2011.11.043

[evad236-B35] Llaurens V , Whibley A, Joron M. Genetic architecture and balancing selection: the life and death of differentiated variants. Mol Ecol. 2017:26(9):2430–2448. 28173627 10.1111/mec.14051

[evad236-B36] Lonsdale J , Thomas J, Salvatore M, Phillips R, Lo E, Shad S, Hasz R, Walters G, Garcia F, Young N, et al. The genotype-tissue expression (GTEx) project. Nat Genet. 2013:45(6):580–585. 23715323 10.1038/ng.2653 PMC4010069

[evad236-B37] Lyon MS , Andrews SJ, Elsworth B, Gaunt TR, Hemani G, Marcora E. The variant call format provides efficient and robust storage of GWAS summary statistics. Genome Biol. 2021:22(1):32. 33441155 10.1186/s13059-020-02248-0 PMC7805039

[evad236-B38] Maclean CA , Chue Hong NP, Prendergast JG. hapbin: an efficient program for performing haplotype-based scans for positive selection in large genomic datasets. Mol Biol Evol. 2015:32(11):3027–3029. 26248562 10.1093/molbev/msv172 PMC4651233

[evad236-B39] McArthur AG , Hegelund T, Cox RL, Stegeman JJ, Liljenberg M, Olsson U, Sundberg P, Celander MC. Phylogenetic analysis of the cytochrome P450 3 (CYP3) gene family. J Mol Evol. 2003:57(2):200–211. 14562963 10.1007/s00239-003-2466-x

[evad236-B40] Nebert D , Dalton T. The role of cytochrome P450 enzymes in endogenous signalling pathways and environmental carcinogenesis. Nat Rev Cancer. 2006:6(12):947–960. 17128211 10.1038/nrc2015

[evad236-B41] Nebert DW , Wikvall K, Miller WL. Human cytochromes P450 in health and disease. Philos Trans R Soc B Biol Sci. 2013:368(1612):20120431. 10.1098/rstb.2012.0431 PMC3538421 23297354

[evad236-B42] Nelson DR , Koymans L, Kamataki T, Stegeman JJ, Feyereisen R, Waxman DJ, Waterman MR, Gotoh O, Coon MJ, Estabrook RW, et al. P450 superfamily: update on new sequences, gene mapping, accession numbers and nomenclature. Pharmacogenetics. 1996:6(1):1–42. 8845856 10.1097/00008571-199602000-00002

[evad236-B43] Nelson DR , Zeldin DC, Hoffman SMG, Maltais LJ, Wain HM, Nebert DW. Comparison of cytochrome P450 (CYP) genes from the mouse and human genomes, including nomenclature recommendations for genes, pseudogenes and alternative-splice variants. Pharmacogenet Genomics. 2004:14(1):1–18. 10.1097/00008571-200401000-00001 15128046

[evad236-B44] Nica AC , Dermitzakis ET. Expression quantitative trait loci: present and future. Philos Trans R Soc B Biol Sci. 2013:368(1620):20120362. 10.1098/rstb.2012.0362 PMC3682727 23650636

[evad236-B45] Qiu H , Taudien S, Herlyn H, Schmitz J, Zhou Y, Chen G, Roberto R, Rocchi M, Platzer M, Wojnowski L. CYP3 phylogenomics: evidence for positive selection of CYP3A4 and CYP3A7. Pharmacogenet Genomics. 2008:18(1):53–66. 18216722 10.1097/FPC.0b013e3282f313f8

[evad236-B46] Rohlfs RV , Swanson WJ, Weir BS. Detecting coevolution through allelic association between physically unlinked loci. Am J Hum Genet. 2010:86(5):674–685. 20381007 10.1016/j.ajhg.2010.03.001 PMC2869012

[evad236-B47] Rojas L , Neumann I, Herrero MJ, Bosó V, Reig J, Poveda JL, Megías J, Bea S, Aliño SF. Effect of CYP3A5*3 on kidney transplant recipients treated with tacrolimus: a systematic review and meta-analysis of observational studies. Pharmacogenomics J. 2015:15(1):38–48. 25201288 10.1038/tpj.2014.38

[evad236-B48] Scott SA , Sangkuhl K, Stein CM, Hulot J-S, Mega JL, Roden DM, Klein TE, Sabatine MS, Johnson JA, Shuldiner AR. Clinical pharmacogenetics implementation consortium guidelines for CYP2C19 genotype and clopidogrel therapy: 2013 update. Clin Pharmacol Ther. 2013:94(3):317–323. 23698643 10.1038/clpt.2013.105 PMC3748366

[evad236-B49] Siewert KM , Voight BF. Detecting long-term balancing selection using allele frequency correlation. Mol Biol Evol. 2017:34(11):2996–3005. 28981714 10.1093/molbev/msx209 PMC5850717

[evad236-B50] Singh O , Sandanaraj E, Subramanian K, Lee LH, Chowbay B. Influence of CYP4F rs2108622 (V433M) on warfarin dose requirement in Asian patients. Drug Metab Pharmacokinet. 2011:26(2):130–136. 21084764 10.2133/dmpk.dmpk-10-rg-080

[evad236-B51] Stark K , Wongsud B, Burman R, Oliw EH. Oxygenation of polyunsaturated long chain fatty acids by recombinant CYP4F8 and CYP4F12 and catalytic importance of Tyr-125 and Gly-328 of CYP4F8. Arch Biochem Biophys. 2005:441(2):174–181. 16112640 10.1016/j.abb.2005.07.003

[evad236-B52] Sudlow C , Gallacher J, Allen N, Beral V, Burton P, Danesh J, Downey P, Elliott P, Green J, Landray M, et al. UK biobank: an open access resource for identifying the causes of a wide range of complex diseases of middle and old age. PLoS Med. 2015:12(3):e1001779. 25826379 10.1371/journal.pmed.1001779 PMC4380465

[evad236-B53] Tavira B , Coto E, Diaz-Corte C, Alvarez V, López-Larrea C, Ortega F. A search for new CYP3A4 variants as determinants of tacrolimus dose requirements in renal-transplanted patients. Pharmacogenet Genomics. 2013:23(8):445–448. 23778326 10.1097/FPC.0b013e3283636856

[evad236-B54] Thompson EE , Kuttab-Boulos H, Witonsky D, Yang L, Roe BA, Di Rienzo A. CYP3A variation and the evolution of salt-sensitivity variants. Am J Hum Genet. 2004:75(6):1059–1069. 15492926 10.1086/426406 PMC1182141

[evad236-B55] Thompson EE , Kuttab-Boulos H, Yang L, Roe BA, Di Rienzo A. Sequence diversity and haplotype structure at the human CYP3A cluster. Pharmacogenomics J. 2006:6(2):105–114. 16314882 10.1038/sj.tpj.6500347

[evad236-B56] Voight BF , Kudaravalli S, Wen X, Pritchard JK, Hurst L. A map of recent positive selection in the human genome. PLoS Biol. 2006:4(3):e72. 16494531 10.1371/journal.pbio.0040072 PMC1382018

[evad236-B57] Wang D , Guo Y, Wrighton SA, Cooke GE, Sadee W. Intronic polymorphism in CYP3A4 affects hepatic expression and response to statin drugs. Pharmacogenomics J. 2011:11(4):274–286. 20386561 10.1038/tpj.2010.28 PMC3248744

[evad236-B58] Weir BS , Cockerham CC. Estimating F-statistics for the analysis of population structure. Evolution. 1984:38(6):1358–1370. 28563791 10.1111/j.1558-5646.1984.tb05657.x

[evad236-B59] Wright WC , Chenge J, Chen T. Structural perspectives of the CYP3A family and their small molecule modulators in drug metabolism. Liver Res. 2019:3(3–4):132–142. 32789028 10.1016/j.livres.2019.08.001 PMC7418881

[evad236-B60] Yasukochi Y , Satta Y. Molecular evolution of the CYP2D subfamily in primates: purifying selection on substrate recognition sites without the frequent or long-tract gene conversion. Genome Biol Evol. 2015:7(4):1053–1067. 25808902 10.1093/gbe/evv056 PMC4419802

[evad236-B61] Yavorska OO , Burgess S. Mendelianrandomization: an R package for performing mendelian randomization analyses using summarized data. Int J Epidemiol. 2017:46(6):1734–1739. 28398548 10.1093/ije/dyx034 PMC5510723

[evad236-B62] Yi M , Cho S-A, Min J, Kim DH, Shin J-G, Lee S-J. Functional characterization of a common CYP4F11 genetic variant and identification of functionally defective CYP4F11 variants in erythromycin metabolism and 20-HETE synthesis. Arch Biochem Biophys. 2017:620:43–51. 28347661 10.1016/j.abb.2017.03.010

[evad236-B63] Zhang JE , Klein K, Jorgensen AL, Francis B, Alfirevic A, Bourgeois S, Deloukas P, Zanger UM, Pirmohamed M. Effect of genetic variability in the CYP4F2, CYP4F11, and CYP4F12 genes on liver mRNA levels and warfarin response. Front Pharmacol. 2017:8:323. 28620303 10.3389/fphar.2017.00323 PMC5449482

