# Supplementary material for: Signatures of Co-evolution and Co-regulation in the CYP3A and CYP4F Genes in Humans
Source: Genome Biol Evol. 2024 Jan 11;16(1):evad236. doi: 10.1093/gbe/evad236 (PMC10805436; doi:10.1093/gbe/evad236)
Supplement: evad236_Supplementary_Data [file evad236_supplementary_data.zip › Figure_S6.pdf]

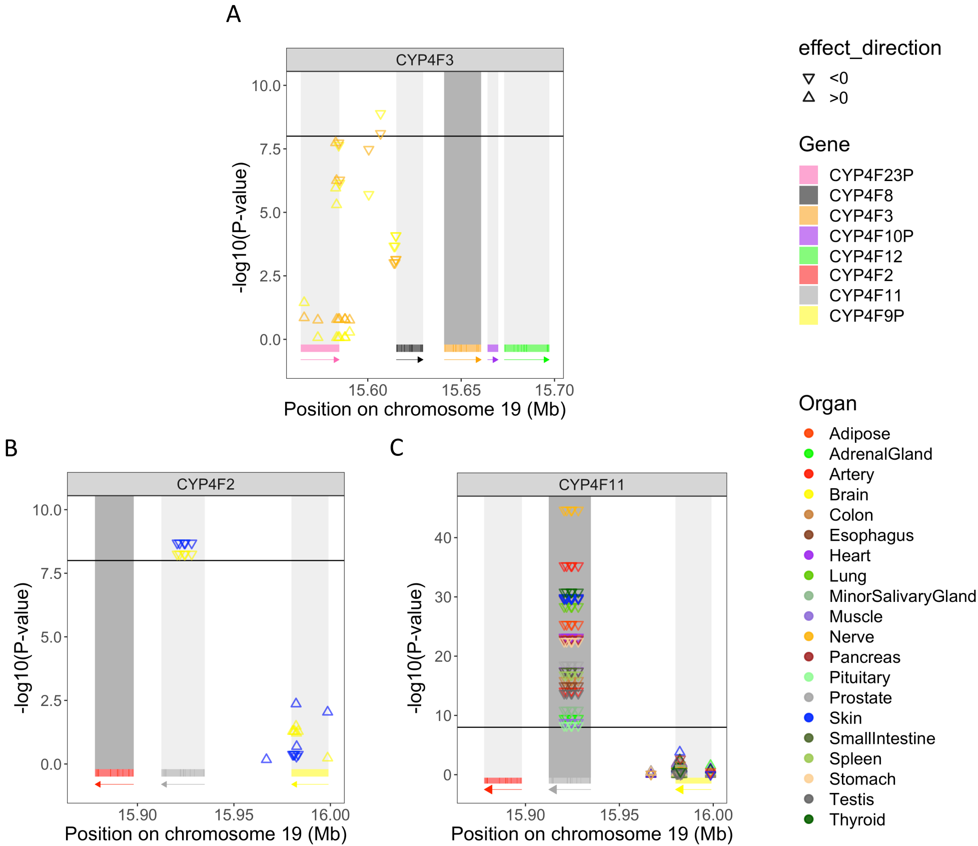
A
CYP4F3
10.0
7.5
5.0
2.5
0.0
-log10(P-value)
15.60
15.65
15.70
Position on chromosome 19 (Mb)
effect_direction
<0
>0
Gene
CYP4F23P
CYP4F8
CYP4F3
CYP4F10P
CYP4F12
CYP4F2
CYP4F11
CYP4F9P
Organ
Adipose
AdrenalGland
Artery
Brain
Colon
Esophagus
Heart
Lung
MinorSalivaryGland
Muscle
Nerve
Pancreas
Pituitary
Prostate
Skin
SmallIntestine
Spleen
Stomach
Testis
Thyroid
B
CYP4F2
10.0
7.5
5.0
2.5
0.0
-log10(P-value)
15.90
15.95
16.00
Position on chromosome 19 (Mb)
C
CYP4F11
40
30
20
10
0
-log10(P-value)
15.90
15.95
16.00
Position on chromosome 19 (Mb)
