# Supplementary material for: Signatures of Co-evolution and Co-regulation in the CYP3A and CYP4F Genes in Humans
Source: Genome Biol Evol. 2024 Jan 11;16(1):evad236. doi: 10.1093/gbe/evad236 (PMC10805436; doi:10.1093/gbe/evad236)
Supplement: evad236_Supplementary_Data [file evad236_supplementary_data.zip › RichardStHilaire_ms_Dec6_2023.NoTracking.Suppl_withrefs.pdf]

13 Supplemental Figures

Figure S1: Positions of genes in the cluster of *CYP3A* (top) and *CYP4F* (bottom) in GRCh37. The direction of the arrow indicate the direction of the gene.

Figure S2: Distribution of Tajima's D values computed on intervals of 1 Kb for each CYP450 gene across each subpopulation in the European population (CEU : Utah residents with Northern and Western European ancestry; TSI : Toscani in Italia; IBS : Iberian populations in Spain; FIN : Finnish in Finland; GBR : British in England and Scotland). The 2.5th percentile is marked by the orange vertical line and the 97.5th percentile is marked by the red vertical line, representing the significance threshold. We kept Tajima's D value for intervals with a high level of mappability (¿90% of interval overlaps high accessibility uniquely mapped regions).

Figure S3: r^2 values between each pairs of SNPs in the A) CYP3A and B) CYP4F cluster for each 1000G population, except YRI (AFR). The genetic distance between the SNPs is in centimorgan (cM). Only r^2 values over the empirical threshold are shown. The empirical distribution is shown with black horizontal lines. Dots are colored according to which genes are involved in the pair.

Figure S4: Recombination map in the CYP3A gene cluster. Each line, with a different line pattern, represents a population and is colored according to the super-population. Each gene and pseudogene are shown below the plot with horizontal line.

Figure S5: Coordinates of each SNP that is in a pair of SNPs with r^2 values in the extremes of the empirical distribution for each subpopulation of 1000G. The displayed SNPs pairs have one SNP in CYP4F12 and the other is in A) CYP4F3 and in B) CYP4F8. We took r^2 values from the previous analysis and filtered to keep only values where one SNP was located in CYP4F12. The graph is generated using the *ggplot*2 library in R. The physical coordinates of each significant Beta signal, identified in the balancing selection analysis, are shown by the vertical red lines, which were created using *geom_vline*. Points were colored according to their respective r^2 values with *scale_color_gradient*.

Figure S6: P-values associated with SNPs under positive selection ($|iHS| \geq 2$) explaining variation of gene expression of A) CYP4F3 B) CYP4F2 and C) CYP4F11. The tested gene is shown in dark gray and the effect size is represented either by a triangle standing on its base or a triangle standing on its point. The threshold, set to 10^{-8}, is represented by the horizontal black line, meaning that a -log10(p-value) >8 is a significant eQTL.

Variant identifier	iHS	Population	Super-population	eQTL
rs74459786	2.00062	JPT	EAS	CYP4F12
	2.09063	STU	SAS	
rs62115147	-2.09205	IBS	EUR	CYP4F3
rs2365175	2.07818	TSI	EUR	CYP4F2, CYP4F11
	2.04181	KHV	EAS	
rs11086013	2.11270	TSI	EUR	CYP4F2, CYP4F11
	2.01056	KHV	EAS	
rs11881793	2.07352	TSI	EUR	CYP4F2, CYP4F11
	2.12697	IBS	EUR	
rs3746154	2.07395	TSI	EUR	CYP4F2, CYP4F11
	2.12768	IBS	EUR	
rs4808413	2.06351	TSI	EUR	CYP4F2, CYP4F11
	2.17967	IBS	EUR	

Table S1: SNPs under positive selection in the CYP4F cluster that are also eQTLs. Each significant SNP is reported with its iHS values ($|iHS| \geq 2$), specific population and RS variant identifier. The gene with differential expression is reported in the eQTL column.

Variant identifier	β score	Population	Super-population
rs644584	72.91370	CEU	EUR
	74.40043	FIN	
	72.64120	GBR	
	77.31849	IBS	
	70.56299	GIH	SAS
rs642322	67.17824	ACB	AFR
	64.12365	ASW	
	77.53712	ESN	
	60.42254	YRI	
	80.95814	CEU	EUR
	79.46902	FIN	
	76.20108	IBS	
	74.54502	GIH	SAS
	75.44200	PJL	
rs74459786	75.65607	ACB	AFR
	59.94361	ASW	
	96.13950	ESN	
	107.11148	GWD	
	89.83249	LWK	
	100.15158	MSL	
	107.11148	GWD	
	85.28441	YRI	
	111.07817	CEU	EUR
	95.36774	FIN	
	115.01944	GBR	
	97.56379	IBS	
	106.51889	CDX	EAS
	83.53252	CHS	
	76.40631	KHV	
	83.65641	BEB	SAS
	74.91468	ITU	
	114.13019	GIH	
	78.72150	PJL	
rs75814017	70.44644	ACB	AFR
	88.88523	ESN	
	100.74132	GWD	
	81.90479	LWK	
	94.13457	MSL	
	76.91697	YRI	
	103.15354	CEU	EUR
	95.98925	FIN	
	115.75484	GBR	
	95.16955	IBS	
	95.82953	TSI	
	101.29256	CDX	EAS
	75.79028	CHS	
	77.93165	BEB	SAS
	73.87701	PJL	

Table S2 continued from previous page

Variant identifier	β score	Population	Super-population
rs73000014	69.60533	ESN	AFR
	74.28322	GWD	
	84.13158	CEU	EUR
	71.43819	FIN	
	78.00721	GBR	
	80.80257	IBS	
	80.54700	TSI	
	83.32819	CDX	EAS
	94.87801	GIH	
rs16980720	60.32447	ACB	AFR
	63.67802	ESN	
	77.61884	CEU	EUR
	79.43404	FIN	
	75.75824	GBR	
	82.68951	IBS	
	75.28558	GIH	SAS
	73.43462	PJL	

Table S2: SNPs under balancing selection in the CYP4F cluster that are also eQTLs of CYP4F12. Each significant SNP is reported with its β values, specific population and RS variant identifier.

Description	Code field	Processing
**Continuous phenotypes suggested by UKb**		
Length of working week for main job	767	
Frequency of travelling from home to job workplace	777	
Age completed full time education	845	
Cooked vegetable intake	1289	
Salad / raw vegetable intake	1299	
Fresh fruit intake	1309	
Dried fruit intake	1319	
Bread intake	1438	
Cereal intake	1458	
Tea intake	1488	
Coffee intake	1498	
Water intake	1528	
Age started wearing glasses or contact lenses	2217	
Age high blood pressure diagnosed	2966	
Age diabetes diagnosed	2976	
Age angina diagnosed	3627	
Age hay fever, rhinitis or eczema diagnosed	3761	
Age asthma diagnosed	3786	
Age heart attack diagnosed	3894	
Age emphysema/chronic bronchitis diagnosed	3992	
Age deep-vein thrombosis (DVT, blood clot in leg) diagnosed	4012	
Age pulmonary embolism (blood clot in lung) diagnosed	4022	
Age stroke diagnosed	4056	
Longest period of depression	4609	

Table S3 continued from previous page

Description	Code field	Processing
Number of depression episodes	4620	
Age glaucoma diagnosed	4689	
Age cataract diagnosed	4700	
Longest period of unenthusiasm / disinterest	5375	
Number of unenthusiastic/disinterested episodes	5386	
Age when loss of vision due to injury or trauma diagnosed	5430	
Age when diabetes-related eye disease diagnosed	5901	
Age macular degeneration diagnosed	5923	
Age other serious eye condition diagnosed	5945	
Hand grip strength (left)	46	
Hand grip strength (right)	47	
Waist circumference	48	
Hip circumference	49	
Standing height	50	
Heel bone ultrasound T-score, manual entry	77	
Heel bone mineral density (BMD) T-score, automated	78	
Heel bone mineral density (BMD) T-score, automated (left)	4106	
Heel bone mineral density (BMD) T-score, automated (right)	4125	
Heel bone mineral density (BMD) T-score, manual entry (left)	4138	
Heel bone mineral density (BMD) T-score, manual entry (right)	4143	
Pulse rate	4194	
Sitting height	20015	
Fluid intelligence score	20016	
Birth weight	20022	
Mean time to correctly identify matches	20023	
Cascot confidence score	20121	
Body mass index (BMI)	21001	
Weight	21002	
Body fat percentage	23099	
Whole body fat mass	23100	
Whole body fat-free mass	23101	
Whole body water mass	23102	
Basal metabolic rate	23105	
Impedance of whole body	23106	
Impedance of leg (right)	23107	
Impedance of leg (left)	23108	
Impedance of arm (right)	23109	
Impedance of arm (left)	23110	
Leg fat percentage (right)	23111	
Leg fat mass (right)	23112	
Leg fat-free mass (right)	23113	
Leg predicted mass (right)	23114	
Leg fat percentage (left)	23115	
Leg fat mass (left)	23116	
Leg fat-free mass (left)	23117	
Leg predicted mass (left)	23118	

Table S3 continued from previous page

Description	Code field	Processing
Arm fat percentage (right)	23119	
Arm fat mass (right)	23120	
Arm fat-free mass (right)	23121	
Arm predicted mass (right)	23122	
Arm fat percentage (left)	23123	
Arm fat mass (left)	23124	
Arm fat-free mass (left)	23125	
Arm predicted mass (left)	23126	
Trunk fat percentage	23127	
Trunk fat mass	23128	
Trunk fat-free mass	23129	
Trunk predicted mass	23130	
Systolic blood pressure, manual reading	93	Values based on the mean of the instance at the first visit
Diastolic blood pressure, manual reading	94	Values based on the mean of the instance at the first visit
Pulse rate (during blood-pressure measurement)	95	Values based on the mean of the instance at the first visit
Pulse rate, automated reading	102	Values based on the mean of the instance at the first visit
Forced vital capacity (FVC)	3062	Values based on the mean of the instance at the first visit
Forced expiratory volume in 1-second (FEV1)	3063	Values based on the mean of the instance at the first visit
Peak expiratory flow (PEF)	3064	Values based on the mean of the instance at the first visit
Diastolic blood pressure, automated reading	4079	Values based on the mean of the instance at the first visit
Systolic blood pressure, automated reading	4080	Values based on the mean of the instance at the first visit
**Blood cells**		
White blood cell (leukocyte) count	30000	
Red blood cell (erythrocyte) count	30010	
Platelet count	30080	
Lymphocyte count	30120	
Monocyte count	30130	
Neutrophill count	30140	
Eosinophill count	30150	
Basophill count	30160	
Nucleated red blood cell count	30170	
Reticulocyte count	30250	
High light scatter reticulocyte count	30300	

Table S3: Continuous phenotypes of the UKb

14 Supplementary text

14.1 D of Tajima additional filtering

To account for the high homology among many CYP450 genes potentially causing problems in callability, we implemented additional filtering steps in our analyses. We used a mappability mask provided by ENCODE (*wgEncodeCrgMapabilityAlign100mer* from UCSC Table Browser) and an accessibility mask (*20140520.combined_mask.autosomes .bed* accessed here). We recalculated Tajima's D using after filtering the VCF using the mappability mask: specifically, we removed all SNPs for which mappability scores were different from 1 in wgEncodeCrgMapabilityAlign100mer (a score of 1 means unique mapping). This was done for each subpopulation, to ensure that population structure does not drive the results. We then kept only Taijma's D values estimated on 1Kb intervals within accessible genomic regions: the mask excludes regions where depth of coverage across all samples was higher or lower than the average depth by a factor of 2-fold. It also excludes sites where >20% of reads had mapping quality of zero. We required that at least 90% of the 1Kb interval overlaps the accessibility mask. All our results remained consistent with our initial observations (Figure S2).

14.2 Pre-processing of GTEx genetic data

Starting from the imputed genotyping dataset, we kept bi-allelic SNPs and removed positions with more than 5% missing genotypes, leaving 100,986 SNPs which were used to perform a PCA using flashPCA2 (Abraham et al., 2017). To retain the non-admixed individuals of European descent, we reduced the dimensionality of the top 10 PCs using the R package `UMAP` (McInnes et al., 2020) (default parameters) to obtain a two dimensional representation of the genetic information contained within those PCs. We identified the largest homogeneous group (self-reported "white") and excluded outlier groups, used only these individuals for the rest of the analyses. We then reran a PCA on this group. We did all subsequent analyses with these 699 individuals. Next we separated each tissue, then removed tissues with fewer than 50 samples, leaving samples from 50 different tissues. We removed in our analyses genes that had fewer than 6 reads in at least 20% of the samples (as recommended by GTEx). We then normalized expression data using limma (TMM normalization) (Ritchie et al., 2015) and voom (Law et al., 2014). We calculated PEER factors (Stegle et al., 2012) on the normalized expressions. The suggested number of PEER factors for the GTEx tissues is 15 for $N < 150$, 30 for $150 \leq N < 250$, 45 for $250 \leq N < 350$, and 60 for $N \geq 350$ (Consortium, 2015).

14.3 Additional analyses on phenotypes

In CYP4F cluster, no SNP under selection was found associated with one of the selected phenotypes. It is possible that the UKb, and more specifically the white British population, is not the appropriate population in which to investigate these selection signals. However, other SNPs in these loci, not found to be under selection in our previous analyses, are associated with forced expiratory volume in 1 second (FEV1) and forced vital capacity (FVC) in the gene *CYP4F2*, and eosinophil count in *CYP4F23P*.

In the *CYP3A* cluster, we identified significant eQTL signals for genes outside the *CYP3A* family, such as *GS1-259H13.2* (ENSG00000244219.6), *ARPC1B* (ENSG00000130429.12) and *ZKSCAN5* (ENSG00000196652.11), located between *CYP3A7* and *CYP3A4* genes and at the 3' end of *CYP3A43* (Figure 6A. These eQTLs are also associated with anthropometric traits, such as fat and height. None of the other SNPs under selection were found to be associated with the expression levels of these genes, meaning that the associations found could be associated with other members of the *CYP3A* family.

In Mendelian randomisation analyses, there were no causal relationship detected between CYP3A5 expression with neither PID (p_{IVW}=0.17), pulse rate (p_{IVW}=0.04, p_{Egger}=0.72,$p_{Intercept}$=0.02), erythrocyte count (p_{IVW}=0.91) and platelet count (p_{IVW}=0.11). For pulse rate, the strongest

signals were located at the 3' end of CYP3A4, suggesting that this outcome could be associated with expression of another gene in the cluster and/or in another tissue, for which we did not have statistical power to detect appropriate eQTL instruments for Mendelian randomisation.

15 Supplementary file

Supplementary file 1. BED file containing the coordinates of the 57 genes used in the manuscript extracted from the UCSC genes table.

References

Abraham, G., Qiu, Y., and Inouye, M. (2017). FlashPCA2: principal component analysis of Biobank-scale genotype datasets. *Bioinformatics*, 33(17):2776–2778.

Consortium, T. G. (2015). The Genotype-Tissue Expression (GTEx) pilot analysis: Multitissue gene regulation in humans. *Science*, 348(6235):648–660. Publisher: American Association for the Advancement of Science Section: Research Article.

Law, C. W., Chen, Y., Shi, W., and Smyth, G. K. (2014). voom: precision weights unlock linear model analysis tools for RNA-seq read counts. *Genome Biology*, 15(2):1–17. Number: 2 Publisher: BioMed Central.

McInnes, J., Healy, J., and Melville, J. (2020). Umap: uniform manifold approximation and projection for dimension reduction. *arXiv:1802.03426 [cs, stat]*.

Ritchie, M. E. et al. (2015). limma powers differential expression analyses for RNA-sequencing and microarray studies. *Nucleic Acids Research*, 43(7):e47–e47.

Stegle, O. et al. (2012). Using probabilistic estimation of expression residuals (PEER) to obtain increased power and interpretability of gene expression analyses. *Nature protocols*, 7(3):500–507.
