# Supplementary figures and images for: Signatures of Co-evolution and Co-regulation in the CYP3A and CYP4F Genes in Humans
Source: Genome Biol Evol. 2024 Jan 11;16(1):evad236. doi: 10.1093/gbe/evad236 (PMC10805436; doi:10.1093/gbe/evad236)

**A**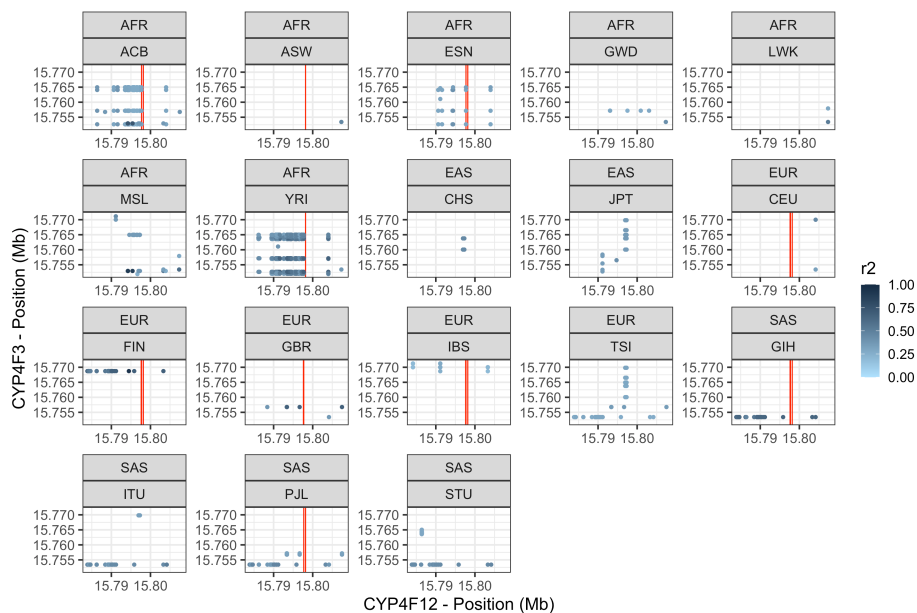**B**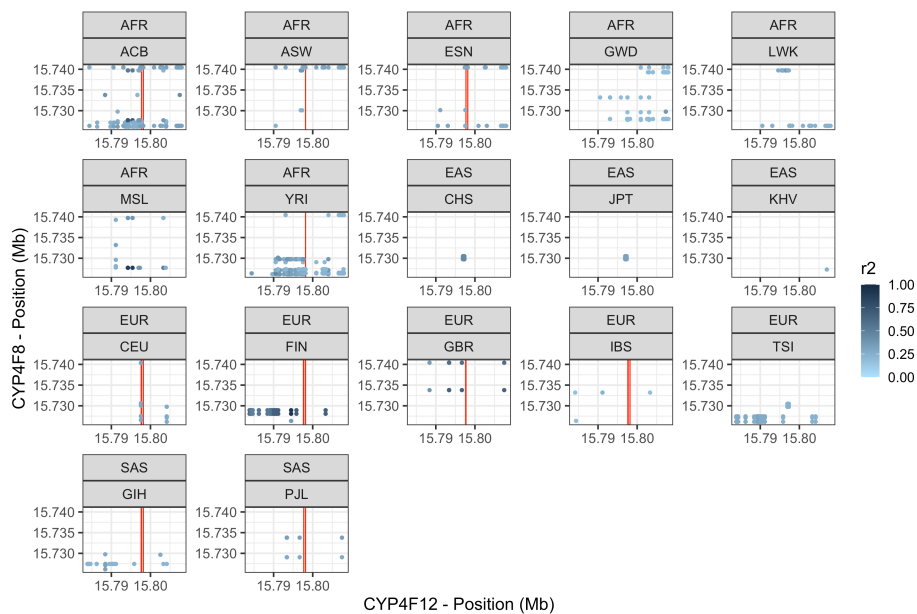

Supplement: evad236_Supplementary_Data [file evad236_supplementary_data.zip › Figure_S5.pdf]

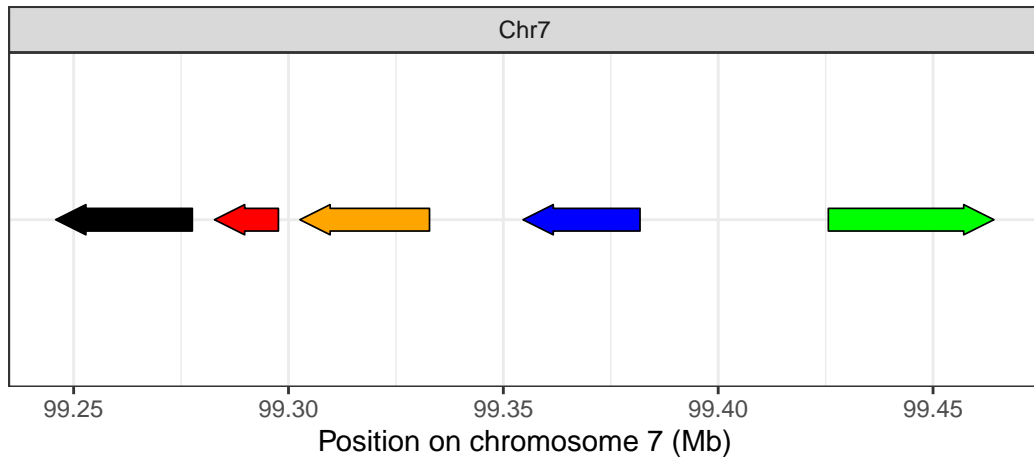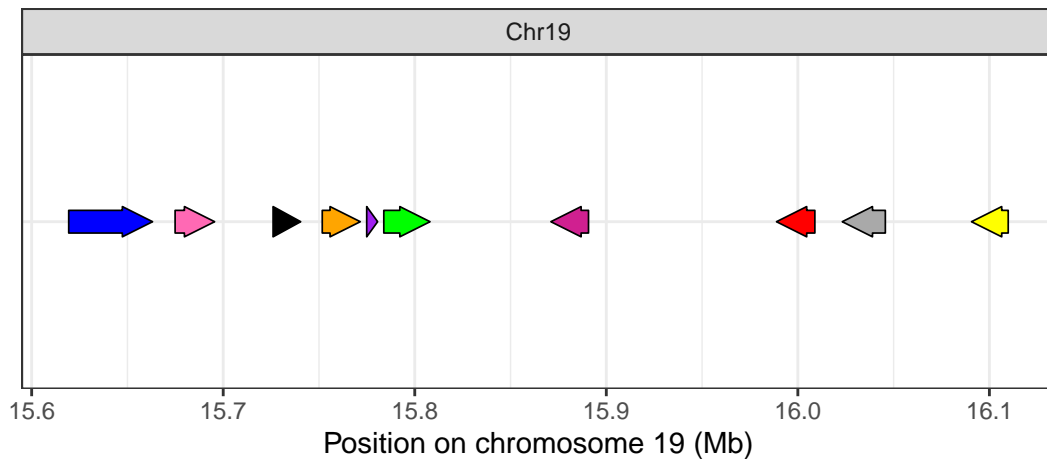

Supplement: evad236_Supplementary_Data [file evad236_supplementary_data.zip › Figure_S1.pdf]

EUR:CEU

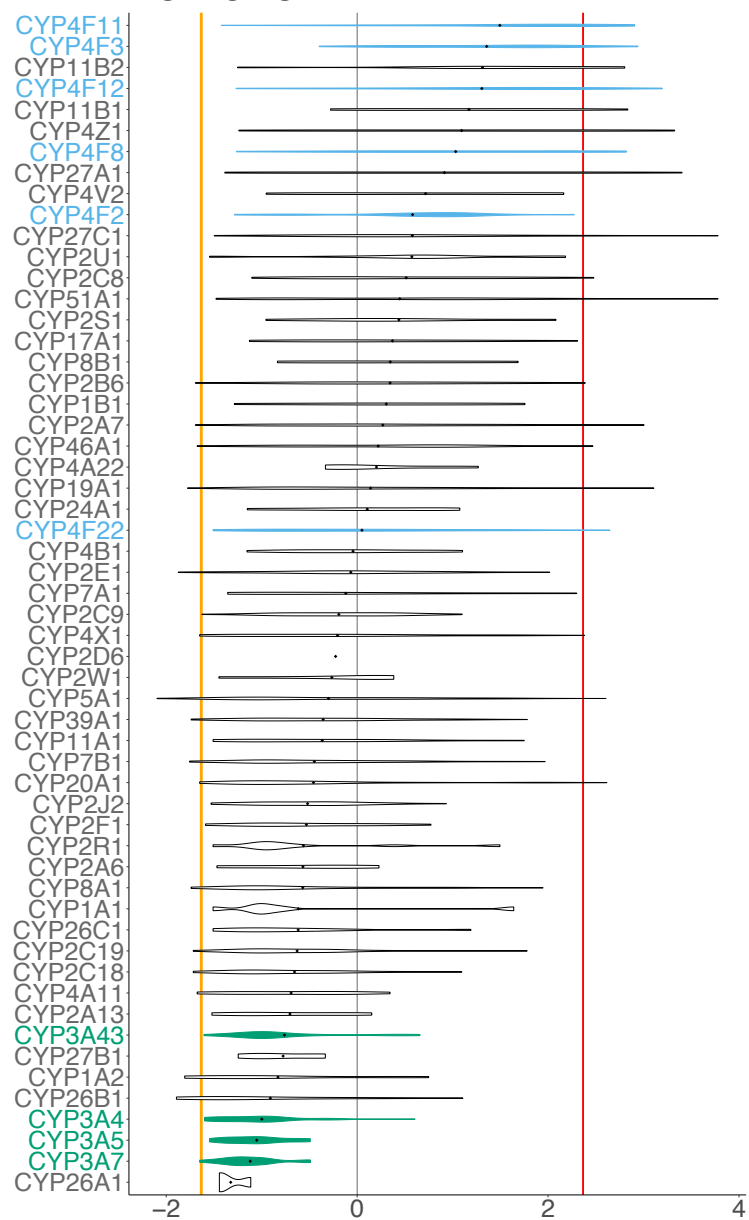

EUR:TSI

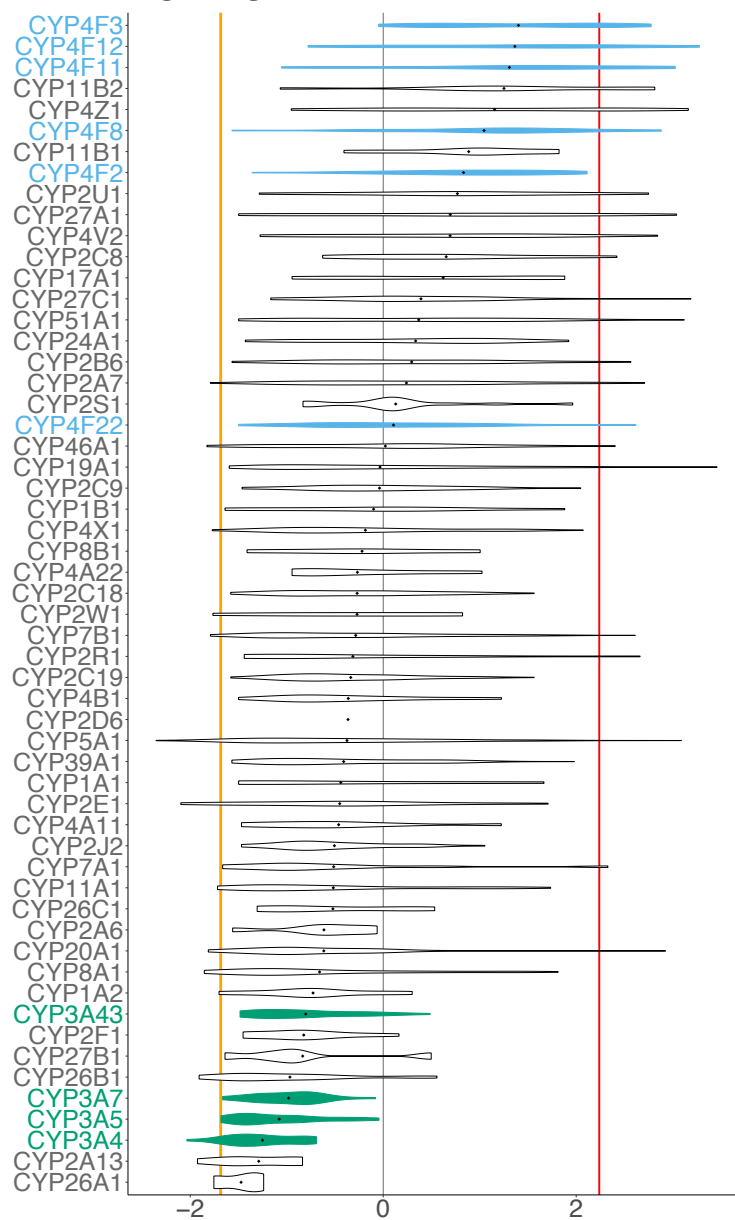

EUR:IBS

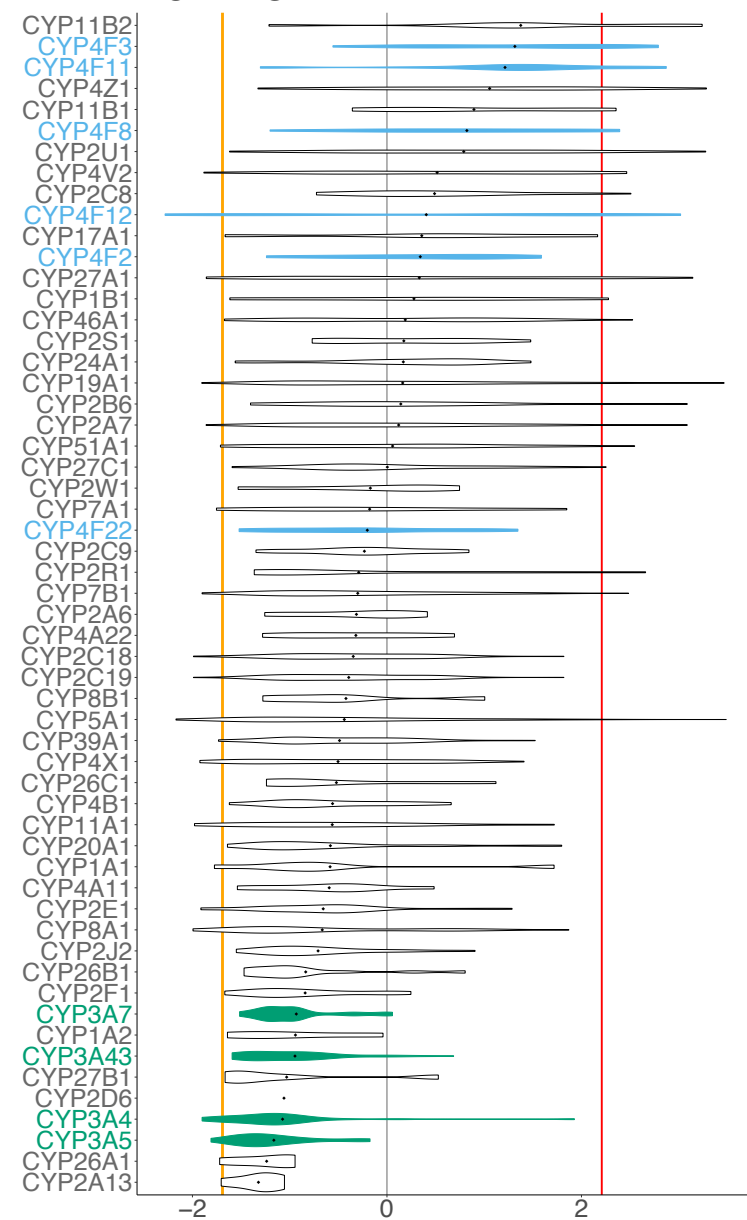

EUR:FIN

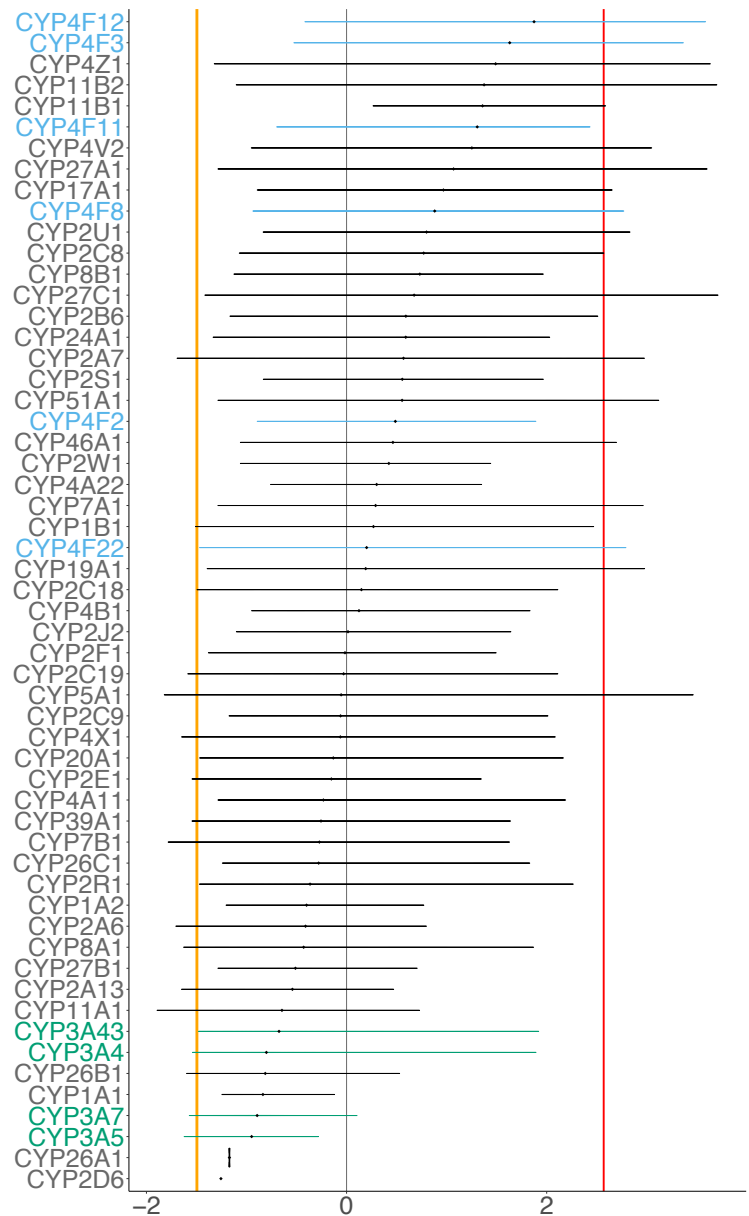

EUR:GBR

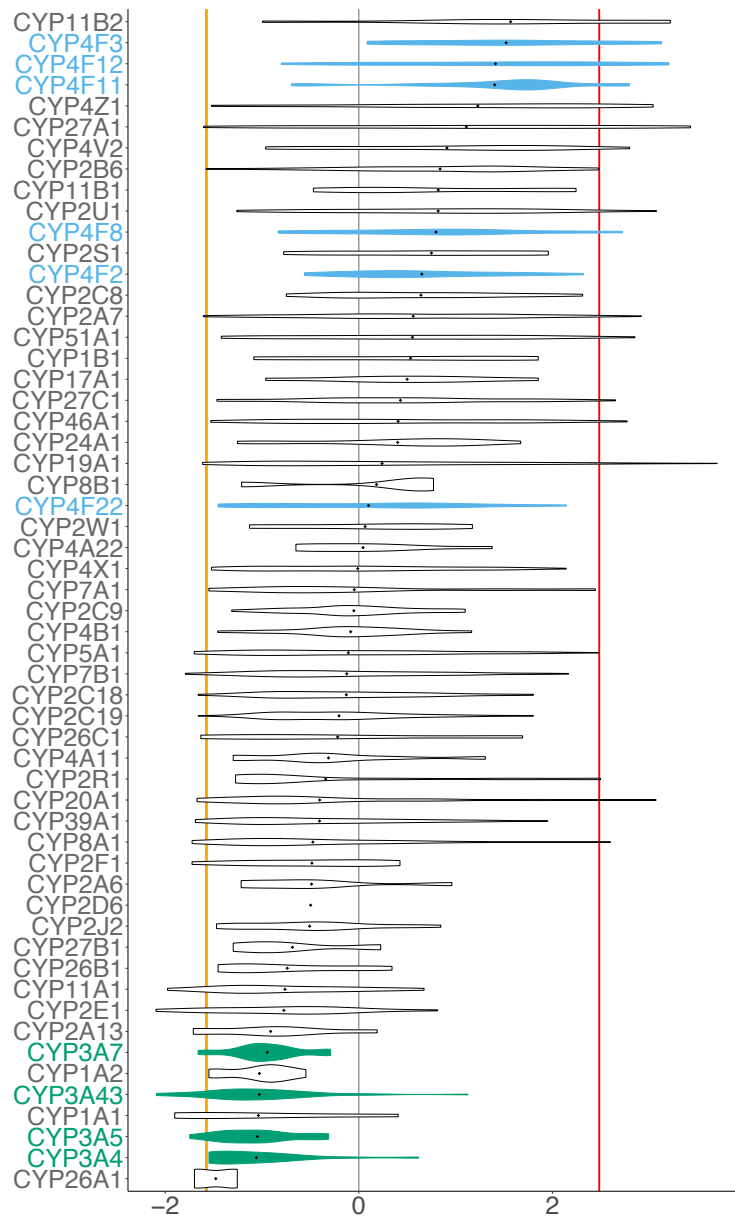

D of Tajima

Supplement: evad236_Supplementary_Data [file evad236_supplementary_data.zip › Figure_S2.pdf]

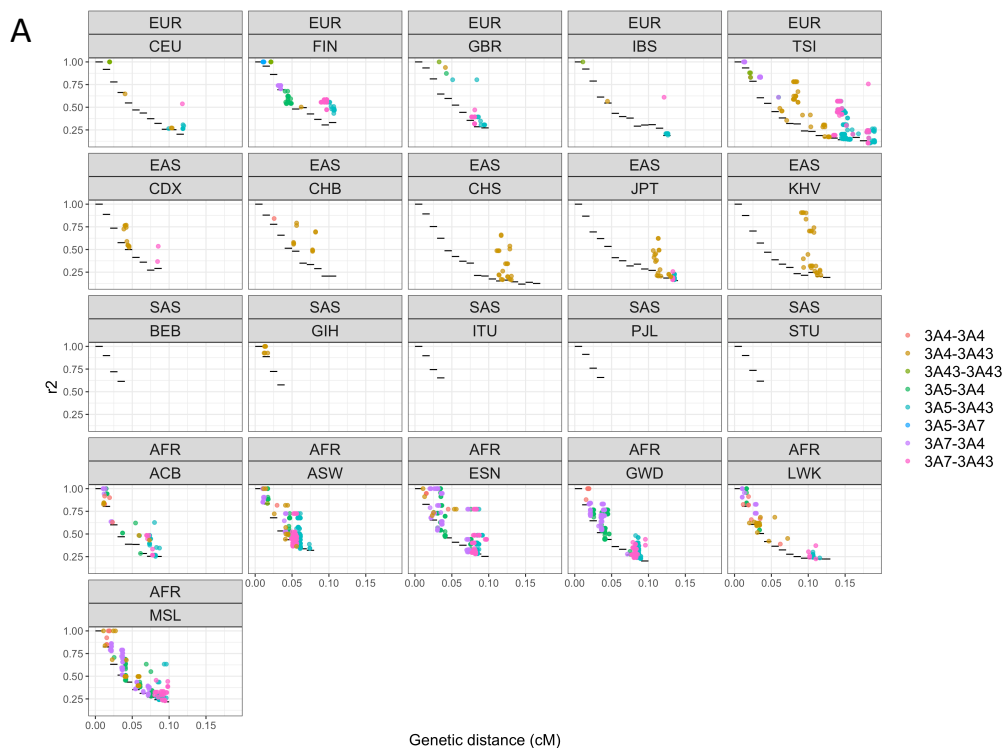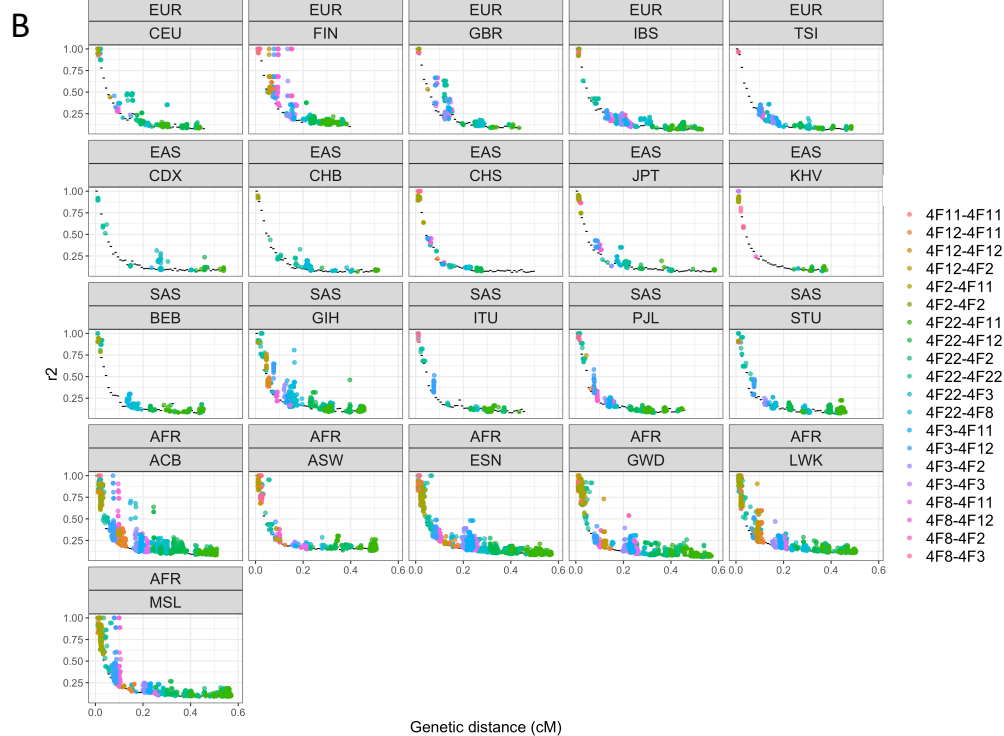

Supplement: evad236_Supplementary_Data [file evad236_supplementary_data.zip › Figure_S3.pdf]

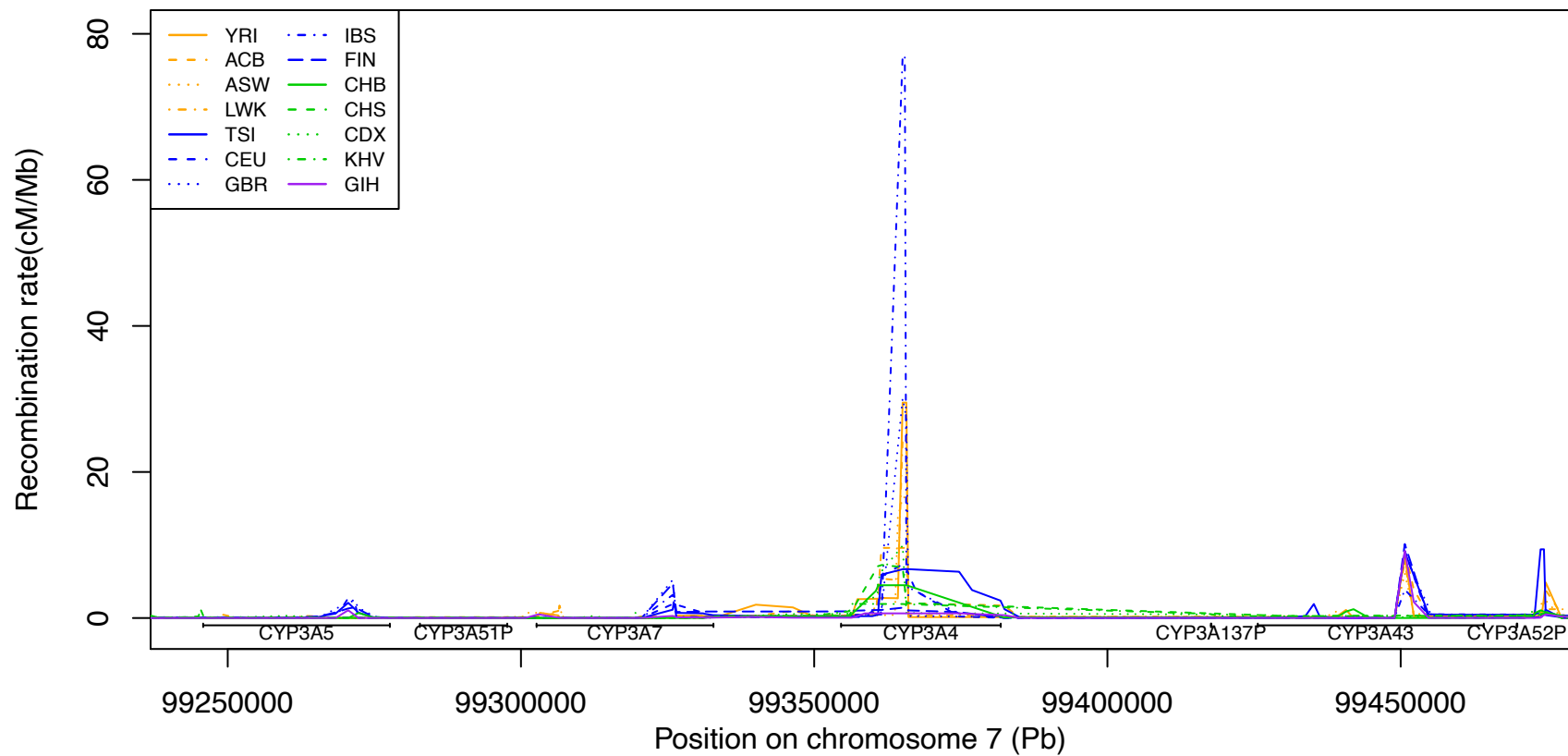

Supplement: evad236_Supplementary_Data [file evad236_supplementary_data.zip › Figure_S4.pdf]
